# Catalytic specificity of the *Lactobacillus plantarum* cystathionine γ-lyase presumed by the crystallographic analysis

**DOI:** 10.1038/s41598-020-71756-7

**Published:** 2020-09-10

**Authors:** Yasuyuki Matoba, Masafumi Noda, Tomoki Yoshida, Kosuke Oda, Yuka Ezumi, Chiaki Yasutake, Hisae Izuhara-Kihara, Narandarai Danshiitsoodol, Takanori Kumagai, Masanori Sugiyama

**Affiliations:** 1 Faculty of Pharmacy, Yasuda Women’s University, Yasuhigashi 6-13-1, Asaminami-ku, Hiroshima, 731-0153 Japan; 2 Graduate School of Biomedical and Health Sciences, Hiroshima University, Kasumi 1-2-3, Minami-ku, Hiroshima, 734-8551 Japan; 3 Faculty of Pharmaceutical Sciences, Hiroshima University, Kasumi 1-2-3, Minami-ku, Hiroshima, 734-8551 Japan

**Keywords:** Biochemistry, Microbiology, Molecular biology, Structural biology

## Abstract

The reverse transsulfuration pathway, which is composed of cystathionine β-synthase (CBS) and cystathionine γ-lyase (CGL), plays a role to synthesize l-cysteine using l-serine and the sulfur atom in l-methionine. A plant-derived lactic acid bacterium *Lactobacillus plantarum* SN35N has been previously found to harbor the gene cluster encoding the CBS- and CGL-like enzymes. In addition, it has been demonstrated that the *L. plantarum* CBS can synthesize cystathionine from *O*-acetyl-l-serine and l-homocysteine. The aim of this study is to characterize the enzymatic functions of the *L. plantarum* CGL. We have found that the enzyme has the high γ-lyase activity toward cystathionine to generate l-cysteine, together with the β-lyase activity toward l-cystine to generate l-cysteine persulfide. By the crystallographic analysis of the inactive CGL K194A mutant complexed with cystathionine, we have found the residues which recognize the distal amino and carboxyl groups of cystathionine or l-cystine. The PLP-bound substrates at the active site may take either the binding pose for the γ- or β-elimination reaction, with the former being the major reaction in the case of cystathionine.

## Introduction

**Figure 1:**
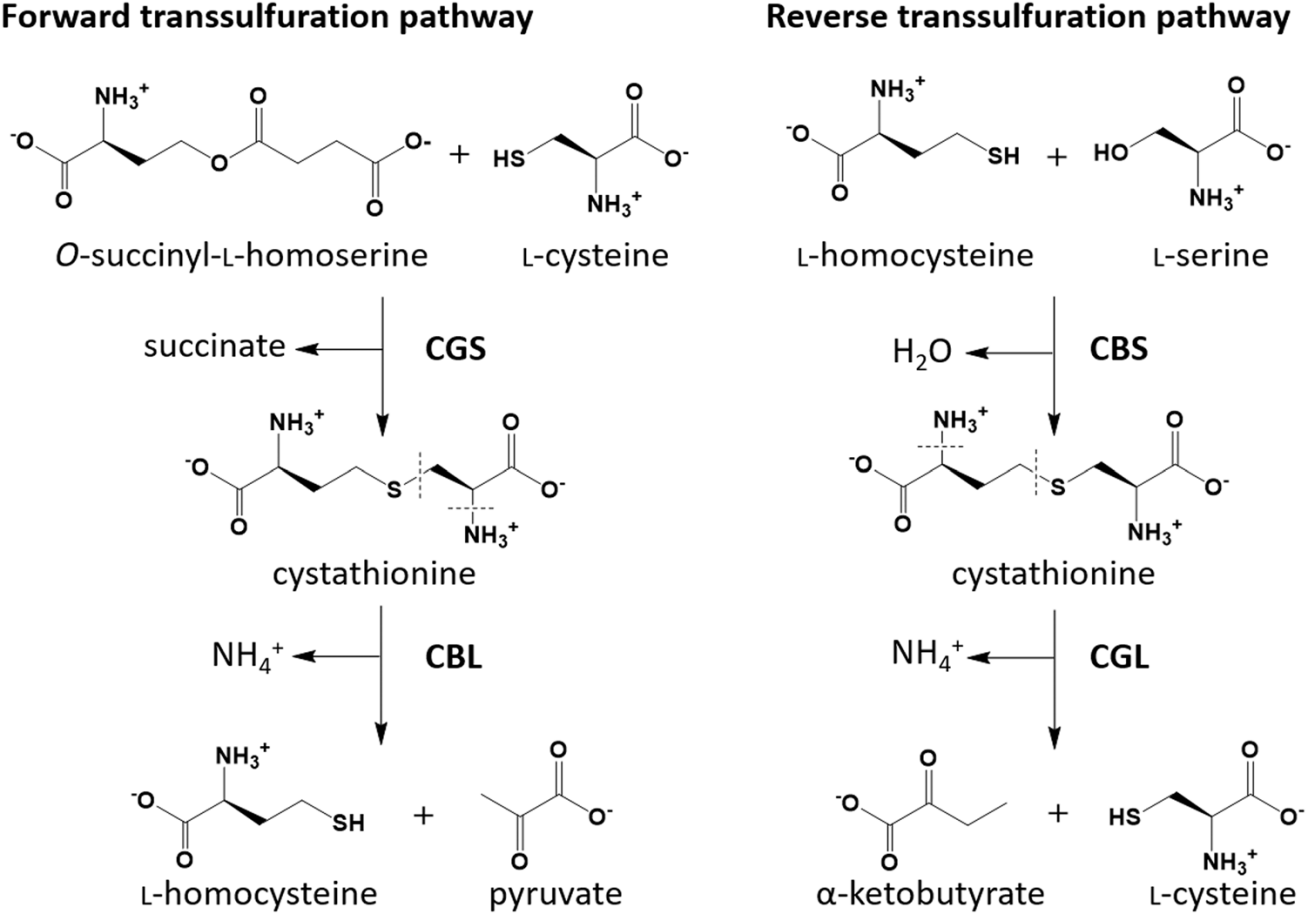
Two metabolic pathways of conversion between l-methionine and l-cysteine. In the forward transsulfuration pathway of *E. coli*, *O*-succinyl-l-homoserine and l-cysteine are linked by cystathionine γ-synthase (CGS), resulting in the generation of cystathionine. Cystathionine β-lyase cleaves the bond between the Cβ and the Sγ atoms to generate l-homocysteine and pyruvate. The l-homocysteine is finally converted to l-methionine by another enzyme. In the reverse transsulfuration pathway of human, l-homocysteine, which is made from l-methionine, and l-serine are linked by cystathionine β-synthase (CBS), resulting in the generation of cystathionine. In the case of *L. plantarum*, *O*-acetyl-l-serine (l-OAS) is used instead of l-serine. Cystathionine γ-lyase (CGL) cleaves the bond between the Cγ and the Sδ atoms to generate l-cysteine and α-ketobutylate. Dotted lines in the figure indicates the covalent bonds to be cleaved by the lyase enzymes. The figure was drawn by ChemDraw (CambridgeSoft).

Transsulfuration pathways play a role to convert between two sulfur-containing amino acid, l-cysteine and l-methionine (Fig. 1)^1^. In eukaryotes, the reverse transsulfuration pathway, which is composed of cystathionine β-synthase (CBS; EC 4.2.1.22) and cystathionine γ-lyase (CGL; EC 4.4.1.1), is involved in the biosynthesis of l-cysteine using l-serine and the sulfur atom in l-methionine. The CBS, which requires pyridoxal 5′-phosphate (PLP) as a cofactor, is an enzyme that catalyzes the formation of cystathionine from l-serine and l-homocysteine^1–3^. The formed cystathionine is decomposed into l-cysteine, ammonium, and α-ketobutyrate by another PLP-dependent enzyme, CGL^1,4,5^. In human, l-homocysteine, which is a nonessential amino acid synthesized from l-methionine, is recognized as a toxic compound^6^. Increased plasma level of l-homocysteine, which is caused by CBS or CGL deficiency, is a risk indicator for thrombosis, atherosclerosis, and vascular disease. Although CBS deficiency causes pathological hyperhomocysteinemia leading to homocystinuria, CGL deficiency, which causes cystathioninuria and mild to moderate homocysteinemia, is essentially a benign disorder^7^.

**Figure 2:**
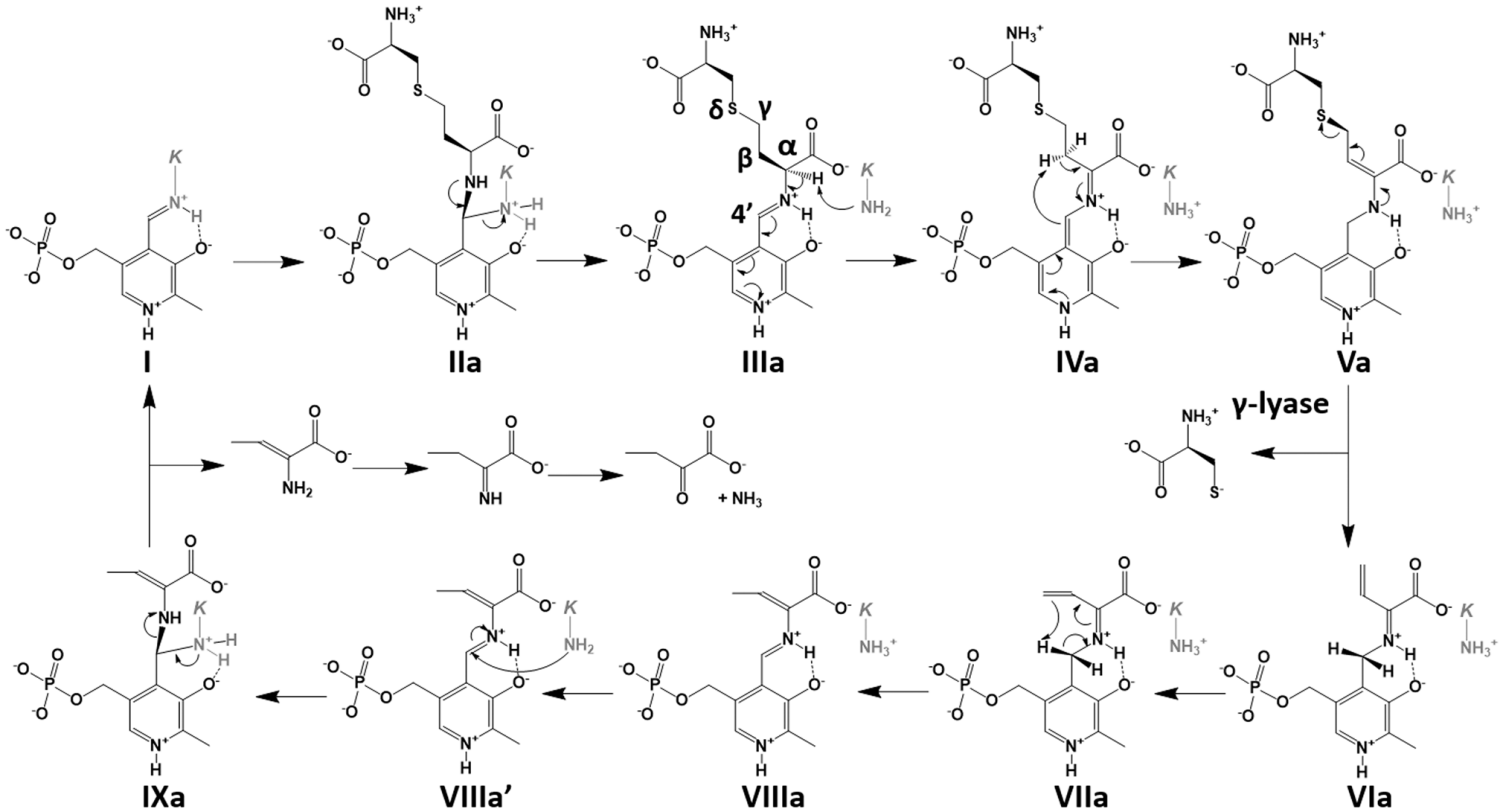
Catalytic mechanism underlying the CGL reaction. Details are described in the text. After the generation of external aldimine formed between PLP and cystathionine (**IIIa**), l-cysteine is released at the γ-lyase step (**Va** to **VIa**). On the other hand, α-aminocrotonate, which is finally degraded to α-ketobutylate and ammonium, is released at the step to regenerate the internal aldimine (**IXa** to **I**). *K* in the figure means the active-site lysine residue. The figure was drawn by ChemDraw.

A proposed catalytic mechanism of CGL, which is based on that of the γ-elimination reaction by the PLP-dependent enzymes^8,9^, is shown in Fig. 2. The initial stages of the CGL reaction occur by the exchange of the ε-amino group of the active-site lysine residue forming an internal aldimine with PLP (**I**) to the α-amino group of cystathionine through the fast formation of the geminal diamine (**IIa**) and its following conversion to the external aldimine (**IIIa**). In cystathionine, there are two amino groups ligating to the carbon atom adjacent to carboxyl group. Therefore, at this stage, cystathionine may be bound in two different modes, in which a sulfur atom is at the γ- or δ-position, although the atom must be set at the latter position to release l-cysteine through the γ-lyase activity of CGL. The structural determinants of CGL to accommodate the cystathionine molecule in the desirable binding mode are under debate^4^. In the external aldimine (**IIIa**), a proton is abstracted from the α-carbon atom of the substrate to the lysine residue, resulting in the formation of a quinonoid intermediate (**IVa**) or its derivative with a carbanion at the C4′ atom in the coenzyme. Subsequent protonation of the C4′ atom and abstraction of the Cβ-proton in the substrate lead to the formation of an enamine intermediate (**Va**) or its derivative with a carbanion at the Cβ atom. Elimination of the l-cysteine would be proceeded by the E1cB mechanism, resulting in the formation of β,γ-unsaturated ketimine (**VIa**). The sequential isomerization (**VIIa**), formations of an α-aminocrotonate (**VIIIa**) and the second geminal diamine intermediates (**IXa**), release of α-aminocrotonate coupled with the regeneration of internal aldimine (**I**), and hydrolysis of the Schiff base in the α-iminobutylate, which is converted from the α-aminocrotonate, generate finally α-ketobutyrate and ammonium. For the catalytic cycle, active-site lysine residue must be deprotonated (**VIIIa′**), prior to the generation of the second geminal diamine (**IXa**).

Besides an important role for the biogenesis of l-cysteine, the human CBS and CGL have been considered to be major physiological resources of H_2_S^10−12^ (Reactions 2, 3, 5, and 8 in Supplementary Fig. S1), which is the third gaseous signaling molecule identified after nitric oxide and carbon monoxide. H_2_S generally plays important roles in the cardiovascular and nervous systems^13^. The H_2_S molecule induces the relaxation of smooth muscle and shows the anti-inflammatory and the cytoprotective effects.

**Figure 3:**
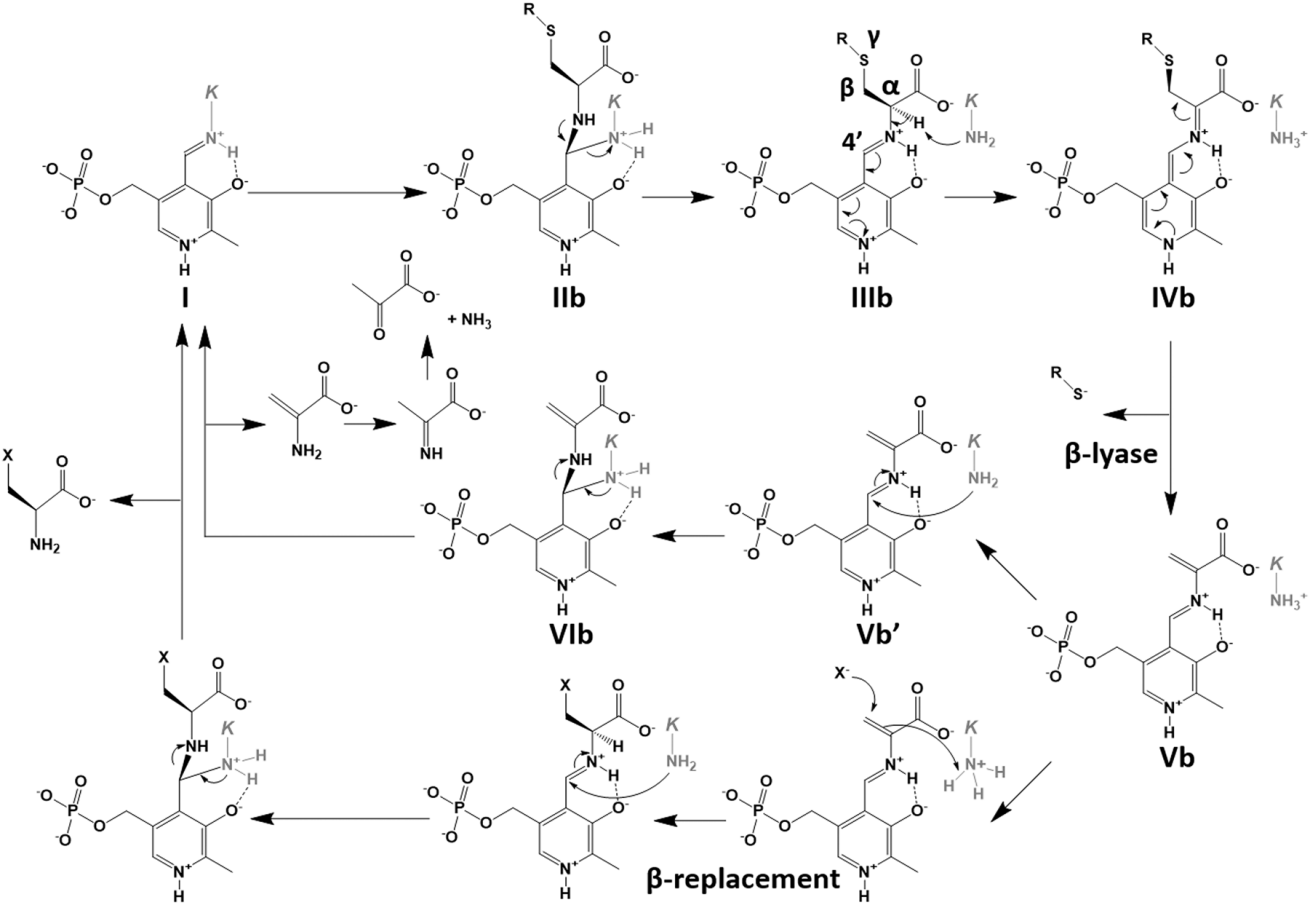
Catalytic mechanism underlying the β-lyase and the β-replacement reactions catalyzed by the CBS and CGL enzymes. Details are described in the text. After the generation of external aldimine formed between PLP and substrate (**IIIb**) in both enzymes, thiol compound is commonly released at the β-lyase step (**IVb** to **Vb**). In the case of CGL, α-aminoacrylate, which is finally degraded to pyruvate and ammonium, is released at the step to regenerate the internal aldimine (**VIb** to **I**). When the substrate is l-cysteine, l-cystine, or cystationine, the R-group is hydrogen, l-cysteine, and l-α-aminobutylate, respectively. In the case of CBS, since the lifetime of the **Vb** intermediate is long enough, the X group can be attached to the Cβ atom by the β-replacement reaction. For the CBS reaction, the X group is l-homocysteine. *K* in the figure means the active-site lysine residue. The figure was drawn by ChemDraw.

In the case of the H_2_S generation catalyzed by CBS^10−12^ (Fig. 3), l-cysteine is used as a substitute for l-serine, which is the first substrate of the enzyme. Elimination of the thiol group by the E2 or E1cb mechanism results in the formation of an α-aminoacrylate intermediate (**Vb**) and H_2_S or its anion. The intermediate may release α-aminoacrylate, which is degraded to ammonia and pyruvate (Reaction 5 in Supplementary Fig. S1), together with the regeneration of internal aldimine (**I**), or may be converted to β-substituted-l-alanine by a β-replacement reaction with a nucleophilic group, such as a thiol group of l-cysteine or l-homocysteine to form lanthionine (Reaction 2 in Supplementary Fig. S1) or cystathionine (Reaction 3 in Supplementary Fig. S1), respectively.

With respect to CGL^12^, H_2_S is mainly generated by two reactions (Reactions 5 and 8 in Supplementary Fig. S1). In the first case (Fig. 3), which is similar to the reaction catalyzed by CBS, H_2_S is released from l-cysteine by β-lyase activity, leading to the formation of an α-aminoacrylate intermediate (**Vb**), which is mainly decomposed into pyruvate and ammonium (Reaction 5 in Supplementary Fig. S1). The intermediate may be formed through geminal diamine (**IIb**), external aldimine (**IIIb**), and quinonoid (**IVb**) intermediates. In the second case, H_2_S is released from l-homocysteine together with the formation of a β,γ-unsaturated ketimine (**VIa** in Fig. 2), followed by the decomposition into α-ketobutyrate and ammonium (Reaction 8 in Supplementary Fig. S1). The reaction scheme should be the same as that of the γ-lyase reaction toward cystathionine (Fig. 2).

In addition, although CGL had been shown to directly catalyze the generation of l-cysteine persulfide from l-cystine by its β-lyase activity^14^ (Reaction 6 in Supplementary Fig. S1), a recent study has demonstrated that CBS can also catalyze the generation^15^ (Reactions 4 and 6 in Supplementary Fig. S1). The biological actions assigned previously to H_2_S may instead be due to the l-cysteine persulfide or its derivatives (hydropersulfides and polysulfides), because the cellular concentrations of the latter compounds are increased by the presence of H_2_S^16,17^.

A plant-derived lactic acid bacterium *Lactobacillus plantarum* harbors a gene cluster composed of CBS- and CGL-encoding open reading frames present on the chromosome, like *Helicobacter pylori* and *Bacillus subtilis*^18−20^. *Bacillus anthracis* and *Pseudomonas aeruginosa* lacking the gene cluster cannot produce H_2_S at a significant level^21^. Moreover, the addition of the inhibitors for human CBS and CGL to *B. anthracis*, *P. aeruginosa*, and *Staphylococcus aureus* was shown to reduce the H_2_S production^21^. Similar to their eukaryotic counterparts, both the CBS and the CGL enzymes in the bacteria may be major sources to generate H_2_S. Interestingly, deletion of the CBS- and CGL-encoding genes and/or addition of the inhibitors toward the enzymes rendered these bacteria highly sensitive to various antibiotics, although the addition of H_2_S suppressed this effect^21^. The mechanism for the H_2_S-mediated antibiotic resistance may be involved in a reduction in the oxidative stress caused by antibiotics, suggesting that the compounds that can inhibit the bacterial CBS or CGL may be useful to enhance the activity of antibiotics.

In the previous study^20^, we have characterized the enzymatic property of the putative CBS from the plant-derived *L. plantarum* SN35N, which has been isolated from pear in Sugiyama’s laboratory^22,23^. The strain produces a large amount of the exopolysaccharide when cultured in the fruit and vegetable juices^24^. Unlike eukaryotic CBSs, the amino acid sequence of the *L. plantarum* CBS is similar to that of *O*-acetyl-l-serine sulfhydrylase (OASS) catalyzing the generation of l-cysteine directly from *O*-acetyl-l-serine (l-OAS) and H_2_S^25,26^ (Reaction 1 in Supplementary Fig. S1). The *L. plantarum* CBS exhibited the activity to synthesize cystathionine using l-OAS (l-OAS-dependent CBS reaction) or l-cysteine (Reaction 3 in Supplementary Fig. S1) as a first substrate and l-homocysteine as a second substrate, together with OASS activity^20^. In particular, the enzyme catalyzes the generation of H_2_S in the presence of l-cysteine and l-homocysteine, according to the synthesis of cystathionine (Reaction 3 in Supplementary Fig. S1). The high affinity toward l-cysteine as a first substrate and high efficiency to use l-homocysteine as a second substrate may be related to the enzymatic ability to generate H_2_S efficiently. In addition, the crystal structure of the *L. plantarum* CBS was determined to clarify the structural basis for the high H_2_S-generation activity^20^.

A compound inhibiting the bacterial biogenesis of H_2_S and l-cysteine persulfide may be used to enhance the activity of antibiotics. To design a new drug, it may be useful to characterize the H_2_S- or l-cysteine persulfide-generating enzymes and to identify the critical residues involved in catalysis. In addition, the degradation of l-cysteine or l-cystine also attracts attention, because reductions in the extracellular concentrations were reported to cause the death of cancer cells due to elevated levels of reactive oxygen species in the cells^27^. For this purpose, a mutant CGL enzyme with high l-cyst(e)ine β-lyase activity was created^27^. In the present study, we characterize the detailed enzymatic properties and determine the crystal structure of the putative CGL enzyme from *L. plantarum* SN35N to provide the structural basis for the reaction specificities. In addition, we have successfully generated a CGL mutant, which has the decreased CGL activity, but the increased l-cyst(e)ine β-lyase activity.

## Results

### Enzymatic properties of the putative CGL enzyme

The putative CGL enzyme from *L. plantarum* SN35N attached with a *C*-terminal His_6_-tag was overexpressed in *Escherichia coli* and purified almost to homogeneity. Judging from the assay using 5,5′-dithiobis-2-nitrobenzoic acid (DTNB)^28^, the enzyme was shown to catalyze the generation of thiol compound(s) from cystathionine (cystathionase activity in Table 1). However, the assay method did not clarify whether the product is l-cysteine or l-homocysteine. CGL produces l-cysteine as a thiol compound in the reverse transsulfuration pathway, while cystathionine β-lyase (CBL) produces l-homocysteine in the forward transsulfuration pathway (Fig. 1). Additionally, since CGL has high sequence similarity to CBL, it is difficult to predict the enzymatic function from the amino acid sequence^1,4^. HPLC analysis after derivatization with 4-(aminosulfonyl)-7-fluoro-2,1,3-benzoxadiazole (ABD-F)^29^ suggested that the concentration of l-cysteine generated was about tenfold higher than that of l-homocysteine. Similarly, the concentration of α-ketobutyrate (by-product formed by CGL) was higher than that of pyruvate (by-product formed by CBL). These results indicate that the purified enzyme acts as CGL in the reverse transsulfuration pathway rather than as CBL (Reaction 7 in Supplementary Fig. S1) in the forward transsulfuration one. The fact that the gene is positioned immediately after the CBS gene also supports this hypothesis.

**Table 1:** Kinetic parameters of *L. plantarum* CGL.

Parameters	Wild type	Y97F
**Cystathionase**		
*k*_cat_ (s^−1^)	0.49 ± 0.01	0.0056 ± 0.0007
*K*_m_ (mM) for cystathionine	0.45 ± 0.01	0.77 ± 0.21
*k*_cat_*/K*_m_ for cystathionine (mM^−1^ s^−1^)	1.1 ± 0.1	0.0072 ± 0.0022
**l-Cysteine β-lyase**		
*k*_cat_ (s^−1^)	0.30 ± 0.03	0.015 ± 0.001
*K*_m_ (mM) for l-cysteine	35 ± 6	0.16 ± 0.03
*k*_cat_*/K*_m_ for l-cysteine (mM^−1^ s^−1^)	0.0086 ± 0.0018	0.096 ± 0.018
**l-Homocysteine γ-lyase**		
*k*_cat_ (s^−1^)	0.081 ± 0.004	0.042 ± 0.020
*K*_m_ (mM) for l-homocysteine	6.3 ± 0.7	1.2 ± 0.8
*K*_I_ (mM) for l-homocysteine	–	0.80 ± 0.51
*k*_cat_*/K*_m_ for l-homocysteine (mM^−1^ s^−1^)	0.013 ± 0.001	0.035 ± 0.027
**l-Cystine β-lyase**		
*k*_cat_ (s^−1^)	0.12 ± 0.01	0.10 ± 0.01
*K*_m_ (mM) for l-cystine	0.12 ± 0.01	0.056 ± 0.009
*k*_cat_*/K*_m_ for l-cystine (mM^−1^ s^−1^)	1.0 ± 0.1	1.8 ± 0.3

Using lead acetate^30^, the H_2_S-generating activity of the *L. plantarum* CGL was analyzed (Table 1). As a result, the enzyme was found to generate H_2_S from l-cysteine or l-homocysteine (l-cysteine β-lyase or l-homocysteine γ-lyase activity in Table 1, respectively), but with roughly 2 orders of magnitude lower catalytic efficiency compared to the canonical CGL activity. On the other hand, using a coupling enzyme l-lactate dehydrogenase and NADH^31^, the enzymatic activity to generate pyruvate from l-cystine was measured (l-cystine β-lyase activity in Table 1). Judging from the *k*_cat_/*K*_m_ values, the catalytic efficiency (1.0 ± 0.1 mM^−1^ s^−1^) was almost the same with that of the cystathionase activity (1.1 ± 0.1 mM^−1^ s^−1^). In addition, cyanolysis analysis^32^ indicated that the reaction mixture contains sulfane sulfur, indicating that l-cysteine persulfide was generated from l-cystine.

In summary, the *L. plantarum* CGL showed γ-lyase activity toward cystathionine and l-homocysteine to generate l-cysteine and H_2_S, respectively. In addition, it demonstrated β-lyase activity toward l-cystine, cystathionine, and l-cysteine to generate l-cysteine persulfide, l-homocysteine, and H_2_S, respectively. In particular, the l-cystine β-lyase and cystathionine γ-lyase activities were notably higher than the others (Table 1). These enzymatic features were also found in eukaryotic CGLs. Although the *L. plantarum* CBS has different characteristics from the eukaryotic CBSs^20^, the cognate CGL is enzymatically similar to the eukaryotic CGLs.

### Crystal structure of the *L. plantarum* CGL

Although crystals of the wild-type *L. plantarum* CGL were obtained, the diffraction data sets were not obtained due to the high mosaicities. The CGL enzymes are known to take open and closed conformations^5^. Structural heterogeneity of the enzyme seems to make structural analysis difficult. To reduce the structural heterogeneity, we tried to crystallize the substrate-bound form. However, if the catalytic lysine residue is present, the reaction after the formation of external aldimine is expected to proceed. Therefore, according to the structural analysis of the *Salmonella* OASS^33^, the catalytic K194 residue of the *L. plantarum* CGL was replaced by alanine. The mutant protein, designated as K194A, was crystallized in the presence of PLP with its substrate (cystathionine) or a substrate analogue (l-serine) (Table 2).

**Table 2:** Data collection and refinement statistics.

Data set	Cystathionine	l-Serine
**Data collection**		
Space group	*C*2	*C*2
Cell dimensions		
* a* (Å)	216.57	217.41
* b* (Å)	200.74	201.15
* c* (Å)	114.88	113.94
* β* (º)	117.21	117.39
Wavelength (Å)	1.0000	1.0000
Resolution (Å)	100–3.10 (3.21–3.10)	100–2.75 (2.85–2.75)
Unique reflection	72,733	112,736
Redundancy^a^	2.0 (2.0)	3.8 (3.8)
Completeness (%)^a^	91.4 (94.2)	99.9 (100)
*R*_merge_ (%)^a, b^	14.2 (43.4)	10.9 (43.6)
*I*/σ^a^	6.0 (1.8)	13.8 (2.8)
**Refinement**		
Resolution (Å)	36.26–3.10	35.68–2.75
Used reflections	72,703	112,712
No. of atoms		
Protein	17,208	17,256
Ligand	174	132
Water/ion	296/15	662/70
*R* (%)	24.57	16.12
*R*_free_ (%)	27.21	20.18
Rms deviations^c^		
Bond length (Å)	0.009	0.007
Bond angle (º)	1.185	0.925
Mean *B*-factor (Å^2^)		
Protein	34.5	51.9
Ligand	35.8	63.5
Water/ion	19.3/28.5	38.5/39.1
Ramachandran plot (%)		
Favored	95.94	97.32
Allowed	3.66	2.68
Disallowed	0.40	–
PDB code	6LE4	6LDO

^a^Values in parentheses are for the highest resolution bin.

^b^*R*_merge_ = Σ|*I* − <*I*>|/Σ*I*, where *I* is the observed intensity and <*I*> is the mean value of *I.*

^c^Rms deviations are calculated by PHENIX^52^.

**Figure 4:**
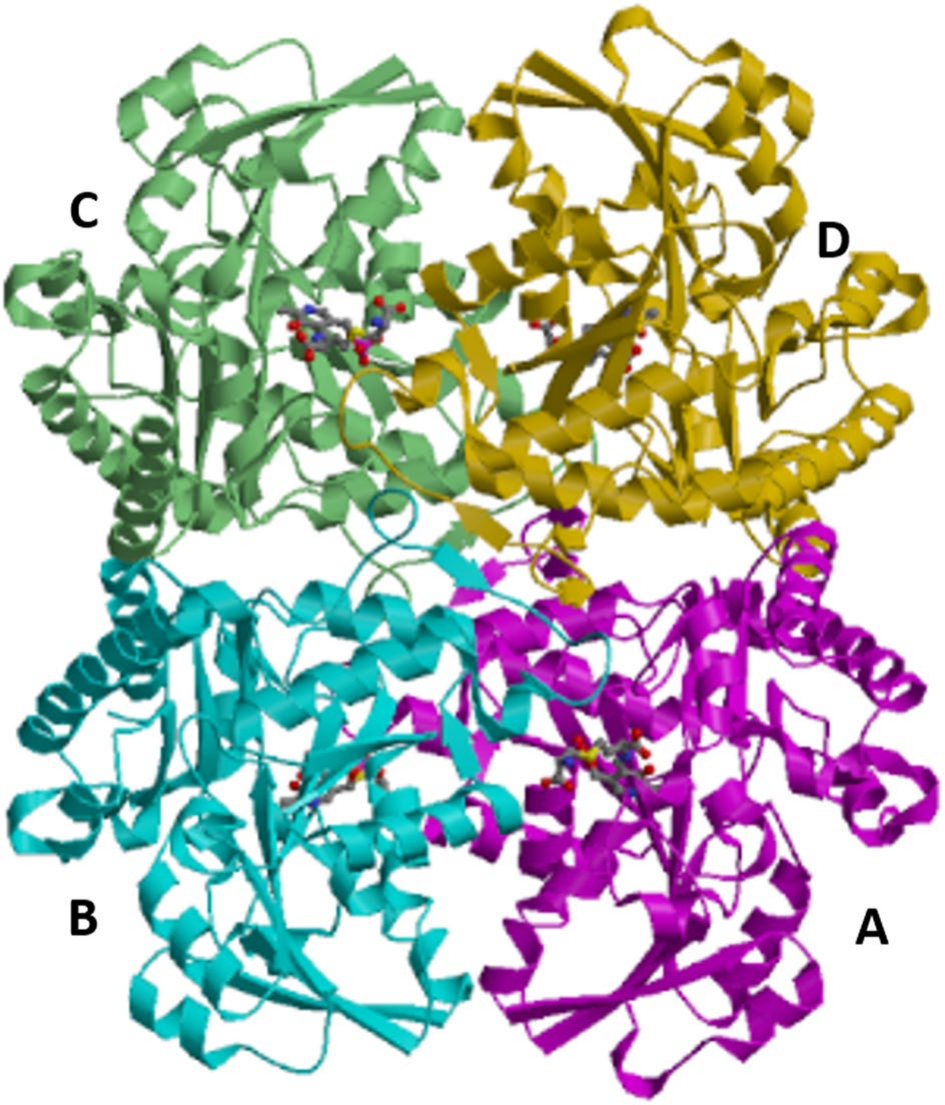
Ribbon diagram of the tetrameric structure of the K194A mutant of *L. plantarum* CGL complexed with external aldimine formed between PLP and cystathionine. Four subunits (A, B, C, and D) are shown in magenta, cyan, light green, and gold. The external aldimine molecules are shown in the stick model. The figure was drawn by PyMOL^55^.

The obtained crystal structures are very similar to the closed forms of other CGL enzymes, including prokaryotic (**PDB IDs**: **4L0O** from *Helicobacter pylori*, **6K1N** from *Stenotrophomonas maltophilia*, **4IYO** from *Xanthomonas oryzae*^34^, and **6KHQ** from *Staphylococcus aureus*^35^) and eukaryotic ones (**PDB IDs**: **1N8P** from yeast^4^ and **2NMP** from human^5^). In addition, the **3QI6** structure from *Mycobacterium ulcerans*^36^ and the **6CJA** structure from *Legionella pneumophila* are deposited as cystathionine γ-synthase (CGS) and CBL, respectively, they are likely to be CGL on the basis of the high structural similarity to the *L. plantarum* CGL. The *L. plantarum* CGL forms a tetramer in the crystal (Fig. 4), like other CGLs.

**Figure 5:**
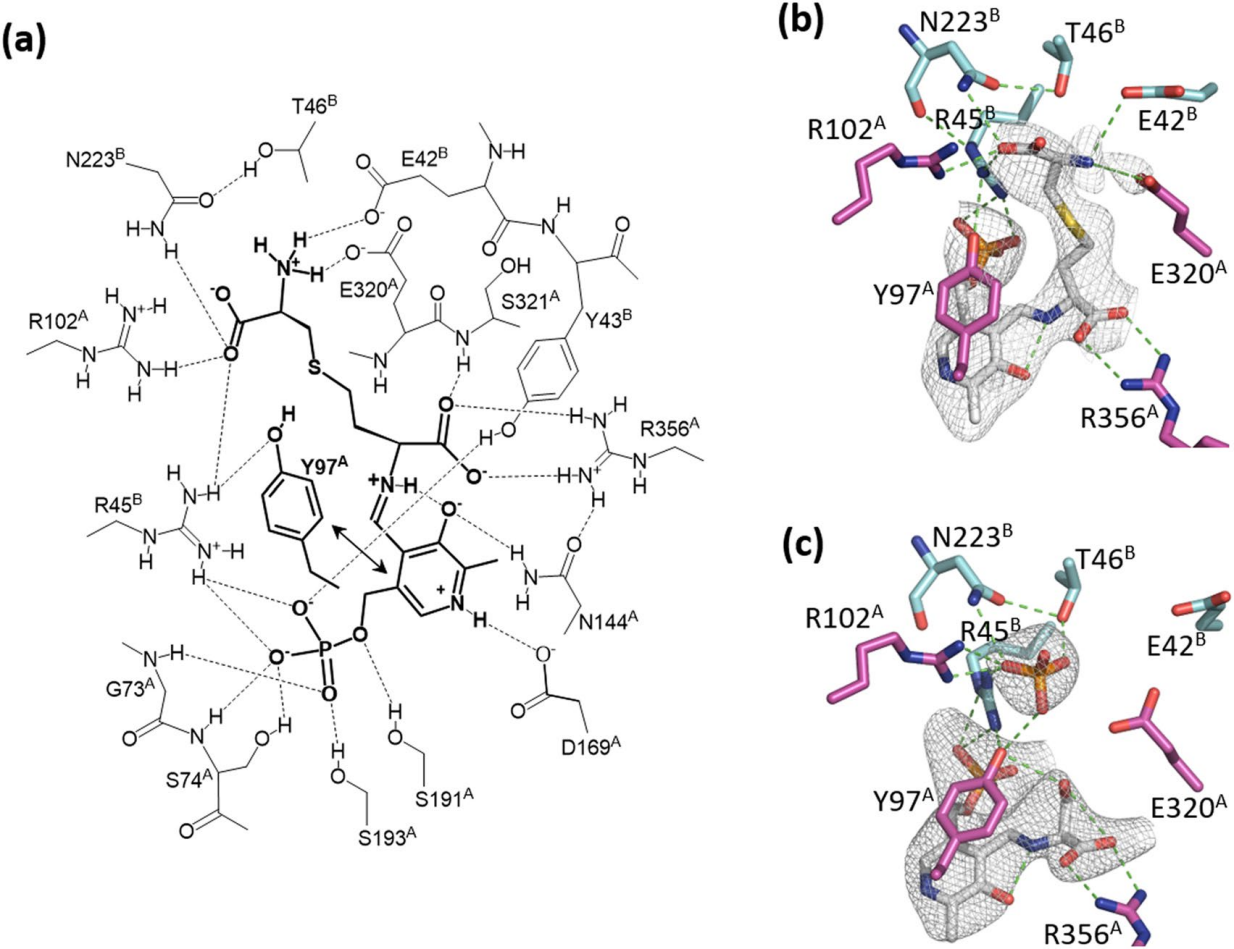
Active site formed between two subunits (A and B) in the crystal structure of the *L. plantarum* CGL. (**a**), Schematic representation of interactions between the residues in CGL and the external aldimine formed between PLP and cystathionine. The pyridine ring in PLP has a stacking interaction with the benzene ring in the Y97^A^ residue. The figure was drawn by ChemDraw. (**b**) or (**c**), Stick model of the active site complexed with cystathionine- or l-serine-bound external aldimine, respectively. Carbon atoms of the external aldimines are shown in gray, whereas those of the residues from subunits A and B are shown in magenta and cyan, respectively. The *F*_o_-*F*_c_ electron density map of the external aldimines are also shown. In **b**, the map was computed after removal of the external aldimine, and contoured at 2.5 σ. In **c**, the map was computed after removals of the external aldimine and phosphate bound at the active site, and contoured at 3 σ. The figures were drawn by PyMOL^55^.

There are four external aldimine molecules bound to the tetramer, and the PLP moieties are anchored by strong hydrogen-bonding interactions with the hydrophilic groups in the residues from neighboring subunits (Figs. 4 and 5a). The pyridine ring of PLP is sandwiched between Y97^A^ and Y43^B^, between Y97^B^ and Y43^A^, between Y97^C^ and Y43^D^, or between Y97^D^ and Y43^C^. The capital letters A, B, C, and D in superscript denote subunit, where the residue is located. Hereafter, we focus on the substrate-binding pocket around one of four PLP molecules, which would be bound to K194^A^. In detail, the phosphate moiety interacts with the backbone nitrogen of G73^A^, the backbone nitrogen and hydroxyl group of S74^A^, and the hydroxyl groups of S191^A^, S193^A^, and Y43^B^, and the guanidino group of R45^B^. In addition, there are two other hydrogen-bonding interactions with PLP: N1 nitrogen and O3 oxygen of PLP interact with the side chains of D169^A^ and N144^A^, respectively. The Y97^A^ residue exhibits aromatic stacking interactions with the pyridine ring of PLP, and its hydroxyl group forms a hydrogen bond with the guanidino group of R45^B^.

Cystathionine was found in the crystal structure as a part of the external aldimine formed with PLP (Fig. 5a,b). The α-carboxylate electrostatically interacts with the side chain of R356^A^ and forms a hydrogen bond with the main-chain nitrogen of S321^A^. On the other hand, the side chain is surrounded by Y97^A^, R102^A^, E320^A^, E42^B^, Y43^B^, R45^B^, T46^B^, and N223^B^. All of the residues interacting with cystathionine are highly conserved among the amino acid sequences of the CGL enzymes, except T46, which is sometimes replaced by serine. The distal carboxyl group in the cystathionine interacts with the guanidino groups of R102^A^ and R45^B^, and the amide nitrogen of N223^B^, whereas the distal amino group interacts with the carboxyl groups of E320^A^ and E42^B^. Since there are many interactions formed between CGL and cystathionine, it is strongly suggested that the compound is a suitable substrate for this enzyme. In addition, the hydroxyl group of the Y97^A^ residue is close to the atoms at the γ- and δ-positions (4.8 ± 0.1 Å and 4.1 ± 0.2 Å, respectively).

Of note, the electron densities at the γ- and δ-positions in cystathionine appears to be almost the same (Fig. 5b), suggesting that cystathionine can be bound in the substrate-binding pocket of CGL in two different modes, in which the sulfur atom occupies the γ- or δ-position. The *L. plantarum* CGL showed both β- and γ-lyase activities toward cystathionine at different catalytic efficiency. Unlike the early proposal^4^, the present crystallographic study suggests that the CGL enzyme is unlikely to have an ability to prioritize one of two different binding modes of cystathionine. However, careful discussions will be required, since our estimation may have large errors due to the low resolution of the crystal structure.

Similarly, l-serine was also found as part of the external aldimine formed with PLP (Fig. 5c). In this case, a phosphate ion contained in the precipitant solution was found at the site for the binding to the distal carboxyl group of cystathionine. The α-carboxylate of the l-serine makes a strong interaction with the CGL enzyme, like that in cystathionine. In addition, the side-chain hydroxyl group may form a hydrogen bond with the side chain of Y97^A^ (the distance is 3.1 ± 0.1 Å), although the electron density of the group is weak.

### Y97F mutation

The Y97 residue is highly conserved among the CGL enzymes and the enzymes structurally similar to CGL but categorized differently, such as l-methionine γ-lyase (MGL)^37^, *O*-acetyl-l-homoserine sulfhydrylase (OAHS)^38^, CGS^39^, and CBL^40^. These enzymes except CBL possess a γ-lyase step in the catalytic mechanism in common. In the MGL reaction, methane thiol is generated from l-methionine as a leaving group, resulting in the formation of a β,γ-unsaturated ketimine intermediate (**VIa** in Fig. 2). On the other hand, in the OAHS or CGS reaction, H_2_S or l-cysteine is attached by Michael addition to the β,γ-unsaturated ketimine intermediate, which is formed after the γ-lyase step to release acetate or succinate to synthesize l-homocysteine or cystathionine, respectively. Since the hydroxyl group of the Y97^A^ residue seems to be close to the atoms at the γ- and δ-positions of the substrates, it may stimulate the β- or γ-lyase step by protonating the leaving group, thereby inhibiting rebound of the leaving group. In the *L. plantarum* CGL, the acidity of the hydroxyl group in the Y97^A^ residue is likely enhanced by the interaction with the R45^B^ residue.

To investigate the effect of the Y97 residue on the kinetics of each reaction, the kinetic parameters of the Y97F mutant were determined (Table 1). The Y97F mutant displayed the extremely weak cystathionase and l-homocysteine γ-lyase activities. Based on kinetic analysis, the weak l-homocysteine γ-lyase activity was due to the substrate inhibition, whereas the weak cystathionase activity was caused by an 88-fold decrease in the *k*_cat_ value. On the other hand, on the basis of the *k*_cat_/*K*_m_ values, the l-cysteine and l-cystine β-lyase activities of the mutant are higher than those of the wild-type (11- and 1.8-fold, respectively). In the case of l-cysteine β-lyase activity, the *K*_m_ value is more lowered by the mutation (220-fold) rather than the *k*_cat_ value (20-fold). On the other hand, both *K*_m_ and *k*_cat_ values of the mutant for the l-cystine β-lyase activity were comparable with those of the wild type (2.1- and 1.2-fold lower than the wild type, respectively).

### Nutritional requirements of *L. plantarum* SN35N

**Figure 6:**
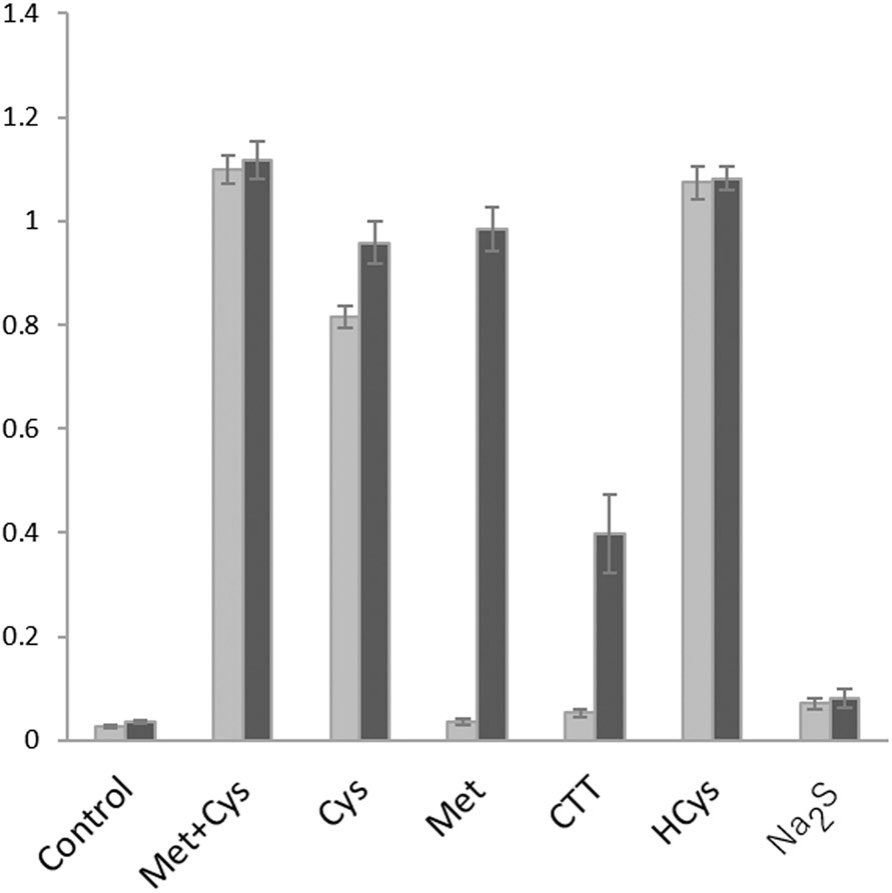
Nutritional requirement of *L. plantarum* SN35N. As a sulfur source, 1.5 mM l-methionine plus 1.5 mM l-cysteine (Met + Cys), 3 mM l-cysteine (Cys), 3 mM l-methionine (Met), 3 mM cystathionine (CTT), 3 mM l-homocysteine (HCys), or 3 mM Na_2_S was supplemented into the chemically defined medium. Gray and black bars indicate the optical densities at 600 nm measured after cultivation for 24 and 48 h, respectively. Each measurement was done in triplicate, and the average and standard deviation are shown in the graph.

We have previously demonstrated that the CBS enzyme from *L. plantarum* SN35N catalyzes the OASS reaction to generate l-cysteine form l-OAS and H_2_S (Reaction 1 in Supplementary Fig. S1)^20^. In addition, the whole genome sequence of the SN35N strain^24^ has shown that the strain possesses a gene encoding OAHS, which catalyzes the generation of l-homocysteine from *O*-acetyl-l-homoserine and H_2_S, like other *L. plantarum* strains^41^. However, the SN35N strain was unable to grow in a synthetic medium where sulfur-containing amino acids were not supplemented (Fig. 6), although the medium contains a possible sulfur source (sulfate). The nutritional requirement of the SN35N strain was consistent with genome analysis that confirms the lack of the enzymes working in the sulfate-reducing pathway^24^. However, the addition of Na_2_S to the medium did not support the bacterial growth, suggesting that the material exhibits toxicity rather than the benefit to supply the sulfur-containing amino acids.

The addition of l-cysteine or l-homocysteine to the medium restored the same growth rate with that observed in the presence of both l-cysteine and l-methionine (Fig. 6). These results suggest that the strain possesses systems to convert l-cysteine to l-methionine via cystathionine and to convert l-methionine to l-cysteine via cystathionine, although other *L. plantarum* strains have been reported to be unable to grow in the presence of either l-cysteine^42^ or l-methionine^43^ as a sole sulfur-containing amino acid. In fact, the strain has a set of genes encoding the enzymes in both transsulfuration pathways (Fig. 1)^24^, like other *L. plantarum* strains^41^.

The addition of l-methionine or cystathionine to the medium permitted the growth of the SN35N strain, although the growth rate was lower when compared with that in the presence of l-cysteine or l-homocysteine (Fig. 6). The low growth rate made by the addition of cystathionine to the medium was probably due to the low solubility of the compound or the low import activity of the strain. On the other hand, the low growth rate made by the addition of l-methionine to the medium may be explained by the low activity of the strain to convert l-methionine to l-homocysteine.

## Discussion

**Figure 7:**
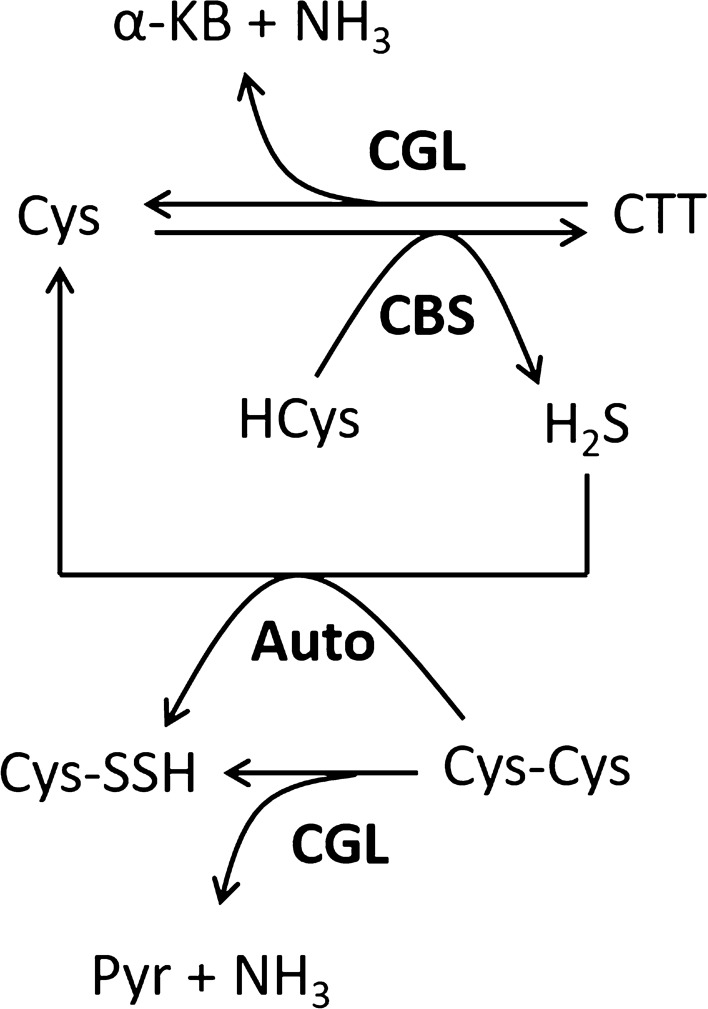
The main pathways for the generations of H_2_S and l-cysteine persulfide in *L. plantarum*. Details are described in the text. Cys, HCys, CTT, Cys-Cys, Cys-SSH, α-KB, and Pyr indicate l-cysteine, l-homocysteine, cystathionine, l-cystine, l-cysteine persulfide, α-ketobutyric acid, and pyruvic acid, respectively.

Enzymatic analysis of CBS in the previous study^20^ and that of CGL in the present study suggested that both enzymes work in the reverse transsulfuration pathway. Participation of the cluster including the CBS and CGL genes in the reverse transsulfuration pathway was also suggested in other lactic acid bacteria^44,45^. In addition, judging from the present (Table 1 and Supplementary Table S1) and previous^20^ studies, both enzymes were shown to have abilities to generate H_2_S and l-cysteine persulfide (Reactions 2–6 and 8 in Supplementary Fig. S1). Based on the catalytic efficiency of the enzymes, the main pathways for the generation of H_2_S and l-cysteine persulfide in *L. plantarum* can be summarized as follows (Fig. 7). The *L. plantarum* CBS generates H_2_S efficiently in the presence of l-cysteine and l-homocysteine along with the synthesis of cystathionine. The consumed l-cysteine will be regenerated from cystathionine by the catalytic activity of the cognate CGL enzyme. The other aspect of these reactions may be the degradation of l-homocysteine coupled with the generation of H_2_S. On the other hand, H_2_S may react with l-cystine, resulting in the non-enzymatic generation of l-cysteine persulfide. In addition, CGL can directly generate l-cysteine persulfide from l-cystine. However, since the concentration of l-cystine is thought to be low in the cell due to the presence of high amounts of reducing agents, l-cysteine persulfide may be generated not only from l-cystine but also by the reaction of l-cysteine with hydropersulfides or polysulfides^16,17^.

The *L. plantarum* CGL can generate l-cysteine and l-homocysteine from cystathionine, although the main product is l-cysteine. Considering the catalytic mechanism of CGL, the γ-lyase reaction would progress easily if the positions of amino nitrogen, carboxyl carbon, Cα, Cβ, and Cγ atoms of cystathionine, which forms the external aldimine with PLP, do not deviate substantially from the same plane (Supplementary Fig. S2). At the same time, the Sδ atom of cystathionine should stick up from the plane for the γ-lyase reaction. On the other hand, the β-lyase reaction would progress if the Cα hydrogen and Sγ atoms in the reverse bound cystathionine are in opposite directions (Supplementary Fig. S3).

In the crystal structure of CGL complexed with cystathionine, the compound is likely bound in two different modes, in which the sulfur atom occupies the γ- or δ-position. The dihedral angle formed among the carboxyl carbon, Cα, Cβ, and Cγ (or Sγ) atoms was 51° ± 6°, whereas the angle formed among the Cα, Cβ, Cγ (or Sγ), and Sδ (or Cδ) atoms was about − 141° ± 1°. Although both dihedral angles deviated from ideal values for the γ-lyase reaction (for example, 0° and − 90° in **Va**, respectively), they would approach the ideal values in accordance with the progress of the catalytic reaction. On the other hand, the former dihedral angle deviated much more from the ideal value for the β-lyase reaction (− 60° and − 90° in **IIIb** and **IVb**, respectively), suggesting difficulty with the reaction. Therefore, it may be concluded that the *L. plantarum* CGL shows a high γ-lyase activity toward cystathionine rather than β-lyase activity, because the enzyme can accommodate it in a suitable conformation for the γ-lyase reaction.

In the course of the CGL reaction, hydrogen atoms bound to the Cα and Cβ atoms in cystathionine must be removed in different steps (**IIIa** to **VIa** and **VIa** to **Va** for the Cα and Cβ hydrogens, respectively). As proposed by many other researchers^8,9^, the amino group of the K194^A^ residue acts as a base to remove the Cα hydrogen. On the other hand, considering the short distance between the Cβ atom of cystathionine and the C4′ atom of PLP in the cystathionine-bound external aldimine, the Cβ hydrogen may directly move to the C4′ atom without a base. After the formation of the enamine intermediate (**Va**), l-cysteine is released by the E1cB reaction mechanism. The reaction may be stimulated by the movement of a proton from the hydroxyl group of the Y97^A^ residue to the thiol group of the leaving l-cysteine. In fact, we observed that the *k*_cat_ value for the cystathionase activity of the Y97F mutant decreased by 88-fold (Table 1). It has been reported that the hydroxyl group of the corresponding tyrosine residue in human CGL forms a covalent bond with the Cγ atom of propargylglycine, a CGL inhibitor^5^. In this case, coupled with the tautomerization of the enamine intermediate (**Va**), a proton from the hydroxyl group of the tyrosine residue may move to the Cδ atom in propargylglycine, resulting in the formation of an allene derivative. Then, the deprotonated hydroxyl nucleophilically attacks the Cγ atom of the derivative.

**Figure 8:**
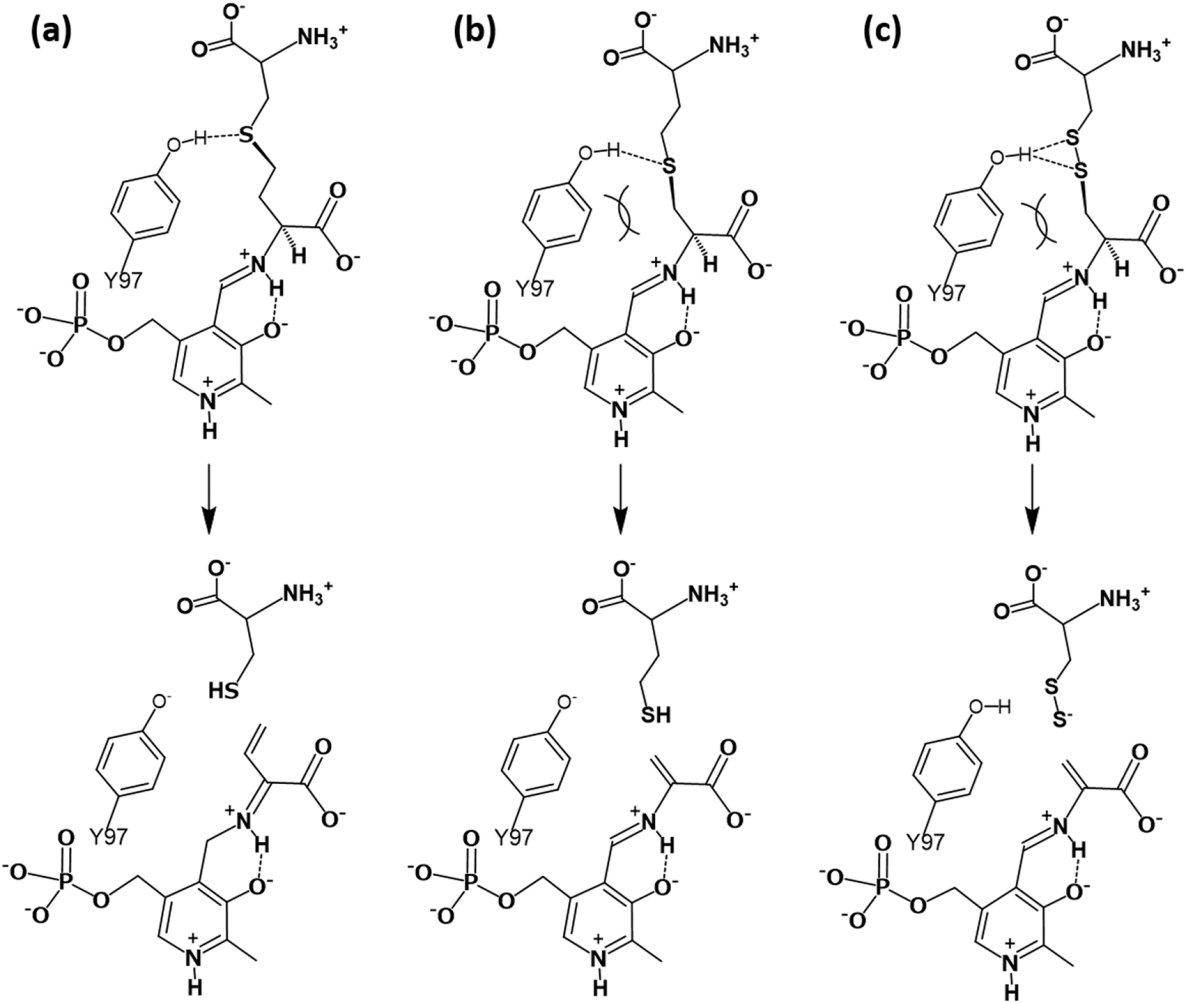
Schematic representation of interactions formed between the Y97 residue in CGL and the substrate bound to PLP. (**a**), Interaction in the CGL reaction. (**b**), Interaction in the CBL reaction. (**c**), Interaction in the l-cystine β-lyase reaction. The hydroxyl group of Y97 seem to make the hydrogen-bonding interactions with the Sγ atom in cystathionine (in **a**), with the Sδ atom in cystathionine (in **b**), and with the Sγ and Sδ atoms in l-cystine (in **c**). The shape of the substrate-binding pocket of CGL is suitable for the CGL reaction. In the case of the CBL and the l-cystine β-lyase reactions, the Y97 may have a difficulty to make the suitable interaction with the substrate due to the close contact. On the other hand, l-cysteine persulfide generated by the l-cystine β-lyase reaction can be released without protonation by the Y97 residue. The figure was drawn by ChemDraw.

The binding pocket for the atoms at the δ and ε positions of cystathionine, which is surrounded by the side chains of residues Y97^A^, E320^A^, and Y43^B^, is very narrow. Therefore, the possible binding poses of cystathionine are restricted. In the major binding pose (Fig. 8a), which is expected on the basis of the current crystal structure complexed with cystathionine-bound external aldimine, the distance from the hydroxyl group of the Y97^A^ residue to the Sδ atom of cystathionine is shorter than that to the Cγ atom, making it possible to form a hydrogen bond between the hydroxyl group of Y97^A^ and the Sδ atom of cystathionine. This is suitable for the γ-lyase reaction.

On the other hand, if cystathionine is accommodated in the pocket in the reverse orientation, the Cδ atom of the substrate comes close to the hydroxyl group of the Y97^A^ residue. This binding pose may cause no reaction. However, as a minor one, cystathionine may be accommodated in a suitable manner for the β-lyase reaction (Fig. 8b), which may reflect the tenfold less CBL activity compared with the CGL activity. The binding pose might be enabled when a hydrogen-bonding interaction is formed between the hydroxyl group of Y97^A^ and the Sγ atoms of cystathionine bound in the reverse orientation, although the interaction may be weak due to the large atomic radius of the sulfur atom. The Y97^A^ may have a difficulty to make such interaction with the reverse bound cystathionine due to the close contact between the benzene ring of the Y97^A^ residue and the Sγ atom of the substrate. The residues that interact with the distal amino and carboxyl groups of cystathionine are not conserved between CGL and CBL^40^. In addition, although the Y97 and Y43 residues of the *L. plantarum* CGL are conserved between CGL and CBL, the residue corresponding to E320 of the *L. plantarum* CGL is tyrosine in CBL. These structural alterations in CBL may make it possible for the reverse bound cystathionine to adopt a binding pose suitable for the β-lyase reaction.

The Y97^A^ residue in the *L. plantarum* CGL is likely to act as an acid to protonate the leaving group. If so, after the elimination reaction, active site of the CGL contains the deprotonated Y97^A^ residue, together with the protonated lysine residue (K194^A^). Protonation of the tyrosine residue and deprotonation of the lysine residue, which are necessary for the next catalytic cycle, may be done at the α-aminocrotonate intermediate (**VIIIa** in Fig. 2) or the α-aminoacrylate intermediate (**Vb** in Fig. 3) (Supplementary Fig. S4).

The *L. plantarum* CGL can also catalyze the H_2_S generation from l-cysteine and l-homocysteine, but with the lower efficiency compared to the canonical reaction. For each reaction, the thiol group of l-cysteine or l-homocysteine may interact with Tyr97^A^, leading to adoption of a suitable binding pose for the β- or γ-lyase reaction, respectively (Supplementary Fig. S5a or S5b, respectively). In the crystal structure of the *L. plantarum* CGL complexed with l-serine as an analog of l-cysteine (Fig. 5c), the dihedral angle formed among carboxyl carbon, Cα, Cβ, and Oγ atoms in the PLP-bound l-serine is − 36° ± 17°, which seemed to be suitable for the β-lyase reaction. The conformation is likely caused by the hydrogen-bonding interaction between the Oγ atom and the hydroxyl group in the Y97^A^ residue (3.1 ± 0.1 Å). The l-cysteine molecule is expected to be bound in a manner similar to l-serine, where the bound l-cysteine molecules undergo the β-lyase reaction to generate H_2_S (Supplementary Fig. S5a).

Based on the *k*_cat_/*K*_m_ values, l-cysteine β-lyase and l-homocysteine γ-lyase activities are 130- and 85-fold lower than the cystathionase activity, respectively (Table 1). In both cases, the *k*_cat_ values were slightly smaller than that for the cystathionase activity (1.6- and 6.0-fold, respectively). On the other hand, *K*_m_ values for both reactions are significantly higher than that for the cystathionase reaction (78- and 13-fold, respectively), being a major reason for the low catalytic efficiencies. The low affinity of CGL toward l-cysteine or l-homocysteine may be due to the absence of the distal amino and carboxyl groups. In addition, the side chain of the Glu320^A^ residue is close to the Cγ and Sδ atoms of the bound cystathionine (4.2 ± 0.2 and 3.5 ± 0.3 Å, respectively) or the Oγ atom of the bound l-serine (4.1 ± 0.1 Å). The l-cysteine or l-homocysteine may be difficult to adopt a suitable binding pose for the respective lyase activity due to the presence of E320^A^, which may form an interaction with the thiol group of substrates (Sγ atom in l-cysteine or Sδ atom in l-homocysteine), and thereby inhibit the desired interaction between the thiol group and Y97^A^ (Supplementary Fig. S5).

The Y97F mutation increased the catalytic efficiency of the l-cysteine β-lyase activity by 11-fold (Table 1). The major reason for the increase in catalytic efficiency of the Y97F mutant was due to the decrease in *K*_m_. In detail, although the *k*_cat_ value for the reaction decreased by 20-fold, the *K*_m_ value decreased more strongly (220-fold). Similarly, mutation of the corresponding tyrosine residue in human CGL to phenylalanine has been reported to increase l-cysteine β-lyase activity^46^. On the other hand, the Y97F mutant hardly showed the l-homocysteine γ-lyase activity due to strong substrate inhibition. Substrate inhibition may occur when l-homocysteine is bound to the pocket where the distal amino and carboxyl groups of cystathionine are accommodated. Although the kinetic parameters determined for the l-homocysteine γ-lyase activity of the mutant are inaccurate because of substrate inhibition, both the *K*_m_ and *k*_cat_ values seem to be reduced (Table 1), as was the case for l-cysteine β-lyase activity.

The reduced *k*_cat_ values for the l-cysteine β-lyase and the l-homocysteine γ-lyase reactions may be due to the lack of the hydroxyl group, which can protonate the leaving hydrogen sulfide anion. On the other hand, the reduced *K*_m_ values suggested that loss of the hydroxyl group from Y97^A^ increased the binding affinity toward l-cysteine and l-homocysteine in spite of the loss of a possible hydrogen-bonding interaction to the substrates. The mutation may expand the substrate-binding pocket and may remove the structural waters present between the Y97^A^ and E320^A^ residues, leading to an increase in the binding affinity toward l-cysteine and l-homocysteine.

Additionally, the *L. plantarum* CGL also catalyzes the β-elimination reaction toward l-cystine to release l-cysteine persulfide (Reaction 6 in Supplementary Fig. S1). l-Cystine is structurally very similar to cystathionine, and the only difference between l-cystine and cystathionine is that the γ-position is occupied by sulfur and carbon atoms, respectively. On the basis of the *k*_cat_/*K*_m_ values (Table 1), the l-cystine β-lyase activity of the *L. plantarum* CGL was similar to the cystathionase activity, which mainly reflects the CGL activity. It is interesting to determine the reason why the CGL enzyme can catalyze the l-cystine β-lyase reaction with similar efficiency to the CGL reaction. As a possible explanation, the Y97^A^ hydroxyl group can form hydrogen bonds with the Sγ and Sδ atoms of l-cystine (Fig. 8c), whereas it forms only one hydrogen bond with the Sγ atom of the reverse bound cystathionine (Fig. 8b). The increased hydrophilic interaction may raise the β-lyase activity more towards l-cystine than towards cystathionine. However, such hydrogen-bonding interactions seem to be difficult due to the close contact between the benzene ring of the Y97^A^ residue and the Sγ atom of l-cystine.

The Y97F mutation decreased slightly both the *K*_m_ and *k*_cat_ values of the l-cystine β-lyase activity (Table 1). A larger and more hydrophobic substrate-binding pocket enhances the binding affinity toward l-cystine and offsets the lack of the possible hydrogen-bonding interactions formed between substrate sulfur atoms and Y97^A^. It should be noted that the *k*_cat_ value was not significantly decreased by the mutation (only 1.2-fold), although the same mutation substantially decreased the *k*_cat_ values of the other reactions (Table 1). The high nucleophilicity of the leaving group, which is generally associated with high basicity, was thought to inhibit the release of the leaving group due to the reverse reaction. The p*K*_a1_ value of H_2_S and p*K*_a_ values of thiol groups in l-cysteine and l-cysteine persulfide were reported to be *ca.* 7.0, 8.3, and 4.3, respectively^47^. On the other hand, the *k*_cat_ value of the mutant for the l-cystine β-lyase reaction was highest among the reactions investigated, followed by the l-cysteine β-lyase and cystathionase reactions (Table 1). The catalytic velocity of the mutant, which lacks the catalytic acid to protonate the leaving group, showed a clear reverse correlation with the basicity of the leaving group, although the nucleophilicity of l-cysteine persulfide was reported to be higher than the value deduced from the p*K*_a_ value due to the α effect^47^. Therefore, it is concluded that l-cystine is a good substrate for both CGL and the Y97F mutant due to the low nucleophilicity of the leaving group.

There have been many mutational studies conducted on eukaryotic CGLs. For example, mutation of the residue in human CGL corresponding to E320 in the *L. plantarum* CGL increased β-lyase activity toward l-cysteine^46^. Double mutations on the residues corresponding to E42 and E320 of the *L. plantarum* CGL increased the β-lyase activities toward both l-cysteine and l-cystine^27^, and triple mutations of residues corresponding to E42, R102, and E320 in the *L. plantarum* CGL increased the MGL activity^48^. On the other hand, mutation of residue(s) in yeast CGL corresponding to E42 and/or E320 of the *L. plantarum* CGL decreased cystathionase activity^49^. Considering that these residues play a role in interacting with distal amino or carboxyl group of cystathionine (Fig. 5a), it is not surprising that the reaction specificity toward cystathionine was decreased by these mutations. Especially, mutation of the residue corresponding to the *L. plantarum* E320 increased the l-cysteine β-lyase activity^27,46^, whereas it decreased the CGL activity^49^. The mutation may remove the possibility to form an undesirable hydrogen bond with the thiol group of l-cysteine, resulting in enhancement of the l-cysteine β-lyase activity of human CGL^27,46^, although this was not true for the yeast CGL^49^. In addition, mutations to increase the size of the pocket for the γ, δ, and ε positions of the substrate and those to change the hydrophobicity of the pocket may alter the substrate and reaction specificity of the enzyme. Another research group succeeded in the creation of an engineered CGL enzyme with high l-cyst(e)ine β-lyase activities to cause the death of cancer cells^27^. In the present study, we demonstrated that the Y97F mutant of *L. plantarum* CGL also has such enzymatic features.

It has been reported that the suppression of H_2_S generation in bacteria made them more sensitive to various antibiotics^21^. The biological actions of H_2_S may be partially due to the increase of l-cysteine persulfide or its derivatives^16,17^. Compounds that specifically inhibit CGL of pathogenic bacteria are expected to suppress the generations of H_2_S and l-cysteine persulfide in the pathogen, and such compounds might be used to enhance the effect of antibiotics against bacteria. Screening of substances that inhibit the bacterial CGL is in progress.

## Materials and methods

### Nutritional requirement analysis

The constitution of chemically defined medium for *L. plantarum* SN35N was designed in reference to a previous study^43^. The medium consisted of 10,000 mg L^−1^ d-glucose, 2000 mg L^−1^ KH_2_PO_4_, 2000 mg L^−1^ ammonium citrate, 5000 mg L^−1^ sodium acetate, 150 mg L^−1^ MgSO_4_, 20 mg L^−1^ MnSO_4_, 10 mg L^−1^ FeSO_4_, 50 mg L^−1^ adenine, 50 mg L^−1^ guanine, 50 mg L^−1^ deoxyguanosine, 50 mg L^−1^ cytidylic acid, 200 mg L^−1^ dl-alanine, 300 mg L^−1^ l-arginine, 200 mg L^−1^ l-asparagine, 200 mg L^−1^ l-aspartic acid, 200 mg L^−1^ l-glutamine, 150 mg L^−1^ l-glutamic acid, 300 mg L^−1^ glycine, 200 mg L^−1^ l-histidine, 200 mg L^−1^ l-isoleucine, 300 mg L^−1^ l-leucine, 300 mg L^−1^ l-lysine, 200 mg L^−1^ l-valine, 200 mg L^−1^ l-phenylalanine, 300 mg L^−1^ l-proline, 300 mg L^−1^ l-serine, 200 mg L^−1^ l-threonine, 200 mg L^−1^ l-tryptophan, 300 mg L^−1^ l-tyrosine, 10 mg L^−1^ biotin, 1 mg L^−1^ nicotinic acid, 1 mg L^−1^ pantothenic acid, 10 mg L^−1^ 4-aminobenzoic acid, 1 mg L^−1^ folic acid, 1 mg L^−1^ pyridoxal, 1 mg L^−1^ riboflavin, 1 mg L^−1^ thiamine, and 1 mg L^−1^ vitamin B_12_. In addition, 1.5 mM l-cysteine plus 1.5 mM l-methionine, 3 mM l-cysteine, 3 mM l-methionine, 3 mM cystathionine, 3 mM l-homocysteine, or 3 mM Na_2_S was supplemented as a sulfur source. The molar concentrations of sulfur atom were set to be equal in all tested conditions. The SN35N strain was grown in MRS broth (Merck) at 37 °C overnight. After centrifugation, the harvested cells were washed three times with saline. Then, the washed cells, the number of which was consistent with that in 20 μL of the overnight culture, was added to 5 mL of the chemically defined medium in a threaded test tube, followed by the static incubation at 37 °C. The optical density at 600 nm was measured after cultivation for 24 and 48 h. Each measurement was done in triplicate.

### Preparation of the *L. plantarum* CGL

Like our previous study for the *L. plantarum* CBS^20^, a gene encoding CGL from *L. plantarum* SN35N was amplified by PCR with KOD DNA polymerase (Toyobo) using a sense primer, 5′-ACATATGAGGACTTTAACAATGAAATTTGAAACCCAA-3′ (*Nde*I site underlined), and an antisense primer, 5′-ACTCGAGATCTGCCTGAATGCTAGCGAAC-3′ (*Xho*I site underlined). After cloning into a pGEM-T vector (Promega), the amplified DNA fragment was digested with *Nde*I and *Xho*I and inserted into a pET-21a( +) vector (Novagen) to generate an expression plasmid for CGL. *E. coli* BL21(DE3) cells harboring an expression vector were grown at 28 °C in Overnight Express Autoinduction System 2 (Novagen). Cells were harvested by centrifugation and disrupted by sonication. *C*-terminal His_6_-tagged CGL was purified by Ni(II) affinity chromatography using His-Bind Resin (Novagen) in accordance with the supplier’s instructions. The fractions containing CGL were dialyzed against a 20 mM Tris–HCl buffer (pH 7.5) containing 0.2 M NaCl, 1 mM EDTA, and 0.2 mM PLP. Supplementation of PLP to the storage buffer for the *L. plantarum* CGL was necessary to keep the clarification and the activity.

### Mutagenesis

Like our previous study for the *L. plantarum* CBS^20^, we used KOD -Plus- Mutagenesis Kit (Toyobo) to generate the Y97F and K194A CGL mutants according to the supplier’s instructions. The mutagenic primers used were as follows: 5′-CGGTGGCACCTTCCGCTTGATC-3′ (Y97F sense), 5′-AAGACATCATTTCCCACAATAATGTG-3′ (Y97F antisense), 5′-GCGTATCTCGGTGGTCACAGTGATG-3′ (K194A sense), and 5′-GGAAGCACTGTGTAAAACAATGTCAACG-3′ (K194A antisense). The underlines in the primers indicate the sequence to produce the substituted codon. To generate an expression plasmid for the CGL variants, that for the wild-type CGL was amplified using the sense and the antisense primers. After the production of the plasmid in *E. coli*, the DNA sequence was analyzed to confirm the introduction of the mutation. Expression and purification of the mutated proteins were done by the same method used for the wild type. However, due to purification of the K194A-mutated CGL, all the buffers used were supplemented with 1 mM PLP and 10 mM l-methionine to prevent the protein precipitation, which was probably caused by the dissociation of the tetramer.

### Product analysis

Analysis of the product from the *L. plantarum* CGL was performed by referencing our previous study for the *L. plantarum* CBS^20^. The reaction mixture, consisting of 100 mM HEPES–KOH (pH 7.5), 5 mM tris(2-carboxyethyl)phosphine-HCl (TCEP-HCl), 5 mM cystathionine, and 280 μg mL^−1^ CGL, was incubated at 37 °C for 10 min. As a control, the same reaction mixture without CGL was used. After incubation for 10 min, the enzymatic reaction was stopped by heating at 95 °C for 5 min, and the precipitate was removed by centrifugation. To detect l-cysteine and l-homocysteine, the supernatant (100 μL) was mixed with equal volume of 0.1 M borate-NaOH buffer (pH 8.0) containing 2 mM EDTA-2NaOH, followed by the addition of double volume of 0.1 M borate-NaOH buffer (pH 8.0) containing 1 mM ABD-F^29^ and 2 mM EDTA-2NaOH. After incubation at 50 °C for 5 min, 300 μL of 0.1 M HCl was added. Then, a 10 μL aliquot of the derivatized sample was injected into an ODS-A column (YMC, 100 × 4.6 mm) equilibrated with 96% solvent A (0.15 M phosphoric acid) and 4% solvent B (acetonitrile containing 0.1% (v/v) trifluoroacetic acid) at 40 °C. The ratio of solvent B was increased linearly from 4 to 15% over 30 min, and from 15 to 100% over the next 30 min. The flow rate was set to 1.0 mL min^−1^. A derivative from the product was detected measuring the absorbance at 380 nm with a multi-wavelength detector.

The reaction mixture, consisting of 100 mM HEPES–KOH (pH 7.5), 5 mM TCEP-HCl, 2 mM substrate (cystathionine, l-cysteine, l-homocysteine, or l-cystine), and 280 μg mL^−1^ CGL, was incubated at 37 °C for 1 h. After adding sulfuric acid to the final concentration of 3.8 mM, the mixture was heated at 95 °C for 5 min, and the precipitate was removed by centrifugation. To detect pyruvic or α-ketobutyric acid, a 10 μL aliquot of the sample was injected into an HPX-87H Aminex ion exclusion column (Bio-Rad, 300 × 7.8 mm) equilibrated with 3.8 mM sulfuric acid at 65 °C. The product was detected measuring the absorbance at 210 nm with a multi-wavelength detector.

### Kinetic analysis

The ability of CGL to release the thiol-containing amino acid (cystathionase activity) was measured in the presence of DTNB^28^. The activity was monitored continuously by measuring absorbance at 412 nm. To determine the kinetic parameters, a reaction mixture (300 μL), consisting of 100 mM HEPES–KOH (pH 7.5), 0.5 mM DTNB, 0.0078–2.0 mM cystathionine, and CGL at an appropriate concentration (50 and 190 μg mL^−1^ in the case of wild-type and the Y97F variant, respectively), was incubated at 37 °C. A molar extinction coefficient of 14,150 M^−1^ cm^−1^ was used for the additives.

The ability of CGL to generate H_2_S from l-cysteine or l-homocysteine was measured in the presence of 0.4 mM lead(II) nitrate^30^. Formation of PbS was monitored continuously by measuring the absorbance at 390 nm. To determine the kinetic parameters, the reaction mixture (300 μL), consisting of 100 mM HEPES–KOH (pH 7.5), 5 mM TCEP-HCl, appropriate concentration ranges of l-cysteine (0.63–80 mM and 0.078–10 mM in the case of wild-type and the Y97F variant, respectively) or l-homocysteine (0.16–20 mM and 0.098–6.3 mM in the case of wild-type and the Y97F variant, respectively), and appropriate concentrations of CGL (50 and 190 μg mL^−1^ in the case of wild-type and the Y97F variant, respectively), was incubated at 37 °C. A molar extinction coefficient of 5500 M^−1^ cm^−1^ was used for lead sulfide.

The ability of CGL to generate l-cysteine persulfide from l-cystine was measured in the presence of l-lactate dehydrogenase and NADH^31^. Formation of pyruvate as a by-product was detected by continuous monitoring of absorbance at 340 nm. To determine the kinetic parameters, the reaction mixture (300 μL), consisting of 100 mM HEPES–KOH (pH 7.5), 0.2 mM NADH, 20 U mL^−1^ l-lactate dehydrogenase, 0.0078–1.0 mM l-cystine, and appropriate concentrations of CGL (50 and 190 μg mL^−1^ in the case of wild-type and the Y97F variant, respectively), was incubated at 37 °C. A molar extinction coefficient of 6220 M^−1^ cm^−1^ was used for NADH.

Each measurement was done in triplicate. When using a single substrate, *k*_cat_ and *K*_m_ values were evaluated using the non-linear least square method by fitting to Eq. 1.

1 $$ v = \frac{{k_{{{\text{cat}}}} \cdot {\text{E}}_{{\text{t}}} \cdot {\text{S}}}}{{K_{{\text{m}}} + {\text{S}}}} $$

In Eq. 1, *v* is the initial velocity, E_t_ and S are the concentrations of the enzyme and substrate, respectively, and *k*_cat_ and *K*_m_ are the catalytic and the Michaelis–Menten constants, respectively.

In the case of l-homocysteine γ-lyase activity of the Y97F-mutated CGL, substrate inhibition was strongly suggested. In this case, data were fitted to Eq. 2.

2 $$ v = \frac{{k_{{{\text{cat}}}} \cdot {\text{E}}_{{\text{t}}} \cdot {\text{S}}}}{{K_{{\text{m}}} + {\text{S}}\left( {1 + \frac{{\text{S}}}{{K_{{\text{I}}} }}} \right)}} $$

The *K*_I_ value is the constant for substrate inhibition.

### Crystallography

Prior to crystallization, the solution of the K194A variant of CGL was concentrated to 9 mg mL^−1^ using Amicon Ultra filters (Millipore). At the same time, 10 mM l-methionine in the preservation buffer was replaced with 2 mM cystathionine or 2 mM l-serine. The CGL crystals were grown using the sitting-drop vapor-diffusion method, with a 1:1 (v/v) ratio of protein solution to precipitant solution. Stick-like crystals were formed within 6 days using a precipitant solution containing 0.4 M KH_2_PO_4_ and 0.4 M Na_2_HPO_4_.

Crystals were flash-frozen before data collection with a cryoprotectant containing 30% (v/v) glycerol. Diffraction intensities of the crystals were collected using synchrotron radiation from BL38B1 at SPring-8 (Hyogo, Japan). X-ray diffraction was measured with a CCD camera at the station, and intensities were integrated and scaled using the HKL2000 program^50^. The tertiary structure of CGL was revealed by the molecular replacement method using the atomic coordinates of the *Helicobacter pylori* CGL (PDB code: **4L0O**, unpublished result) as a search model and the Molrep program in the CCP4 program suite^51^. The asymmetric unit contains 4 monomers forming a tetramer and 2 monomers forming another tetramer together with 2 crystallographic symmetry related monomers. The model was refined by the PHENIX program^52^ with non-crystallographic symmetry restraints. A subset of 5% of the reflections was used to monitor the free *R* factor (*R*_free_)^53^. After the refinement, the model was revised using the COOT program^54^. Details of data collection and refinement statistics are shown in Table 2. The atomic coordinates and structure factors of the *L. plantarum* CGL complexed with cystathionine and l-serine have been deposited in the Protein Data Bank with accession codes **6LE4** and **6LDO**, respectively.

## Supplementary information

Supplementary Information.

## References

[1] Aitken SM, Lodha PH, Morneau DJ (2011). The enzymes of the transsulfuration pathways: active-site characterizations. Biochim. Biophys. Acta.

[2] Miles EW, Kraus JP (2004). Cystathionine β-synthase: structure, function, regulation, and location of homocystinuria-causing mutations. J. Biol. Chem..

[3] Aitken SM, Kirsch JF (2005). The enzymology of cystathionine biosynthesis: strategies for the control of substrate and reaction specificity. Arch. Biochem. Biophys..

[4] Messerschmidt A, Worbs M, Steegborn C, Wahl MC, Huber R, Laber B, Clausen T (2003). Determinants of enzymatic specificity in the Cys-Met-metabolism PLP-dependent enzymes family: crystal structure of cystathionine γ-lyase from yeast and intrafamiliar structure comparison. Biol. Chem..

[5] Sun Q, Collins R, Huang S, Holmberg-Schiavone L, Anand GS, Tan CH, van den Berg S, Deng LW, Moore PK, Karlberg T, Sivaraman J (2009). Structural basis for the inhibition mechanism of human cystathionine γ-lyase, an enzyme responsible for the production of H_2_S. J. Biol. Chem..

[6] Castro R, Rivera I, Blom HJ, Jakobs C, Tavares de Almeida I (2006). Homocysteine metabolism, hyperhomocysteinaemia and vascular disease: an overview. J. Inherit. Metab. Dis..

[7] Finkelstein JD (2006). Inborn errors of sulfur-containing amino acid metabolism. J. Nutr..

[8] Brzović P, Holbrook EL, Greene RC, Dunn MF (1990). Reaction mechanism of *Escherichia coli* cystathionine γ-synthase: direct evidence for a pyridoxamine derivative of vinylglyoxylate as a key intermediate in pyridoxal phosphate dependent γ-elimination and γ-replacement reactions. Biochemistry.

[9] Sato D, Shiba T, Karaki T, Yamagata W, Nozaki T, Nakazawa T, Harada S (2017). X-Ray snapshots of a pyridoxal enzyme: a catalytic mechanism involving concerted [1,5]-hydrogen sigmatropy in methionine γ-lyase. Sci. Rep..

[10] Singh S, Ballou DP, Banerjee R (2011). Pre-steady-state kinetic analysis of enzyme-monitored turnover during cystathionine β-synthase-catalyzed H_2_S generation. Biochemistry.

[11] Yadav PK, Banerjee R (2012). Detection of reaction intermediates during human cystathionine β-synthase-monitored turnover and H_2_S production. J. Biol. Chem..

[12] Singh S, Padovani D, Leslie RA, Chiku T, Banerjee R (2009). Relative contributions of cystathionine β-synthase and γ-cystathionase to H_2_S biogenesis *via* alternative trans-sulfuration reactions. J. Biol. Chem..

[13] Szabó C (2007). Hydrogen sulphide and its therapeutic potential. Nat. Rev. Drug Discov..

[14] Yamanishi T, Tuboi S (1981). The mechanism of the l-cystine cleavage reaction catalyzed by rat liver γ-cystathionase. J. Biochem..

[15] Ida T, Sawa T, Ihara H, Tsuchiya Y, Watanabe Y, Kumagai Y, Suematsu M, Motohashi H, Fujii S, Matsunaga T, Yamamoto M, Ono K, Devarie-Baez NO, Xian M, Fukuto JM, Akaike T (2014). Reactive cysteine persulfides and S-polythiolation regulate oxidative stress and redox signaling. Proc. Natl. Acad. Sci. USA.

[16] Alvarez L, Bianco CL, Toscano JP, Lin J, Akaike T, Fukuto J (2017). The chemical biology of hydropersulfides and related species: possible roles in cellular protection and redox signaling. Antioxid. Redox. Signal..

[17] Fukuto JM, Ignarro LJ, Nagy P, Wink DA, Kevil CG, Feelisch M, Cortese-Krott MM, Bianco CL, Kumagai Y, Hobbs AJ, Lin J, Ida T, Akaike T (2018). Biological hydropersulfides and related polysulfides - a new concept and perspective in redox biology. FEBS Lett..

[18] Hullo MF, Auger S, Soutourina O, Barzu O, Yvon M, Danchin A, Martin-Verstraete I (2007). Conversion of methionine to cysteine in *Bacillus subtilis* and its regulation. J. Bacteriol..

[19] Doherty NC, Shen F, Halliday NM, Barrett DA, Hardie KR, Winzer K, Atherton JC (2010). In *Helicobacter pylori*, LuxS is a key enzyme in cysteine provision through a reverse transsulfuration pathway. J. Bacteriol..

[20] Matoba Y, Yoshida T, Izuhara-Kihara H, Noda M, Sugiyama M (2017). Crystallographic and mutational analyses of cystathionine β-synthase in the H_2_S-synthetic gene cluster in *Lactobacillus plantarum*. Protein Sci..

[21] Shatalin K, Shatalina E, Mironov A, Nudler E (2011). H_2_S: a universal defense against antibiotics in bacteria. Science.

[22] Jin H, Higashikawa F, Noda M, Zhao X, Matoba Y, Kumagai T, Sugiyama M (2010). Establishment of an in vitro Peyer's patch cell culture system correlative to in vivo study using intestine and screening of lactic acid bacteria enhancing intestinal immunity. Biol. Pharm. Bull..

[23] Zhao X, Higashikawa F, Noda M, Kawamura Y, Matoba Y, Kumagai T, Sugiyama M (2012). The obesity and fatty liver are reduced by plant-derived *Pediococcus pentosaceus* LP28 in high fat diet-induced obese mice. PLoS ONE.

[24] Noda M, Shiraga M, Kumagai T, Danshiitsoodol N, Sugiyama M (2018). Characterization of the SN35N strain-specific exopolysaccharide encoded in the whole circular genome of a plant-derived *Lactobacillus plantarum*. Biol. Pharm. Bull..

[25] Rabeh WM, Cook PF (2004). Structure and mechanism of *O*-acetylserine sulfhydrylase. J. Biol. Chem..

[26] Mozzarelli A, Bettati S, Campanini B, Salsi E, Raboni S, Singh R, Spyrakis F, Kumar VP, Cook PF (2011). The multifaceted pyridoxal 5’-phosphate-dependent *O*-acetylserine sulfhydrylase. Biochim. Biophys. Acta.

[27] Cramer SL, Saha A, Liu J, Tadi S, Tiziani S, Yan W, Triplett K, Lamb C, Alters SE, Rowlinson S, Zhang YJ, Keating MJ, Huang P, DiGiovanni J, Georgiou G, Stone E (2017). Systemic depletion of l-cyst(e)ine with cyst(e)inase increases reactive oxygen species and suppresses tumor growth. Nat. Med..

[28] Winther JR, Thorpe C (2014). Quantification of thiols and disulfides. Biochim. Biophys. Acta.

[29] Imai K, Uzu S, Toyo'oka T (1989). Fluorogenic reagents, having benzofurazan structure, in liquid chromatography. J. Pharm. Biomed. Anal..

[30] Willhardt I, Wiederanders B (1975). Activity staining of cystathionine-β-synthetase and related enzymes. Anal. Biochem..

[31] Williamson JR, Corkey BE (1969). Assays of intermediates of the citric acid cycle and related compounds by fluorometric enzyme methods. Methods Enzymol..

[32] Wood JL (1987). Sulfane sulfur. Methods Enzymol..

[33] Burkhard P, Tai CH, Ristroph CM, Cook PF, Jansonius JN (1999). Ligand binding induces a large conformational change in *O*-acetylserine sulfhydrylase from *Salmonella typhimurium*. J. Mol. Biol..

[34] Ngo HP, Cerqueira NM, Kim JK, Hong MK, Fernandes PA, Ramos MJ, Kang LW (2014). PLP undergoes conformational changes during the course of an enzymatic reaction. Acta Crystallogr. D Biol. Crystallogr..

[35] Lee D, Jeong S, Ahn J, Ha NC, Kwon AR (2019). Crystal structure of bacterial cystathionine γ-lyase in the cysteine biosynthesis pathway of *Staphylococcus aureus*. Crystals.

[36] Clifton MC, Abendroth J, Edwards TE, Leibly DJ, Gillespie AK, Ferrell M, Dieterich SH, Exley I, Staker BL, Myler PJ, Van Voorhis WC, Stewart LJ (2011). Structure of the cystathionine γ-synthase MetB from *Mycobacterium ulcerans*. Acta Crystallogr. Sect. F Struct. Biol. Cryst. Commun..

[37] Motoshima H, Inagaki K, Kumasaka T, Furuichi M, Inoue H, Tamura T, Esaki N, Soda K, Tanaka N, Yamamoto M, Tanaka H (2000). Crystal structure of the pyridoxal 5'-phosphate dependent l-methionine γ-lyase from *Pseudomonas putida*. J. Biochem..

[38] Tran TH, Krishnamoorthy K, Begley TP, Ealick SE (2011). A novel mechanism of sulfur transfer catalyzed by *O*-acetylhomoserine sulfhydrylase in the methionine-biosynthetic pathway of *Wolinella succinogenes*. Acta Crystallogr. D Biol. Crystallogr..

[39] Clausen T, Huber R, Prade L, Wahl MC, Messerschmidt A (1998). Crystal structure of *Escherichia coli* cystathionine γ-synthase at 1.5 Å resolution. EMBO J..

[40] Clausen T, Huber R, Laber B, Pohlenz HD, Messerschmidt A (1996). Crystal structure of the pyridoxal-5'-phosphate dependent cystathionine β-lyase from *Escherichia coli* at 1.83 Å. J. Mol. Biol..

[41] Liu M, Nauta A, Francke C, Siezen RJ (2008). Comparative genomics of enzymes in flavor-forming pathways from amino acids in lactic acid bacteria. Appl. Environ. Microbiol..

[42] Wegkamp A, Teusink B, de Vos WM, Smid EJ (2010). Development of a minimal growth medium for *Lactobacillus plantarum*. Lett. Appl. Microbiol..

[43] Saguir FM, de Nadra MC (2007). Improvement of a chemically defined medium for the sustained growth of *Lactobacillus plantarum*: nutritional requirements. Curr. Microbiol..

[44] Bogicevic B, Irmler S, Portmann R, Meile L, Berthoud H (2011). Characterization of the *cysK2-ctl1-cysE2* gene cluster involved in sulfur metabolism in *Lactobacillus casei*. Int. J. Food Microbiol..

[45] Wüthrich D, Irmler S, Berthoud H, Guggenbühl B, Eugster E, Bruggmann R (2018). Conversion of methionine to cysteine in *Lactobacillus paracasei* depends on the highly mobile *cysK-ctl-cysE* gene cluster. Front. Microbiol..

[46] Huang S, Chua JH, Yew WS, Sivaraman J, Moore PK, Tan CH, Deng LW (2010). Site-directed mutagenesis on human cystathionine-γ-lyase reveals insights into the modulation of H_2_S production. J. Mol. Biol..

[47] Cuevasanta E, Lange M, Bonanata J, Coitiño EL, Ferrer-Sueta G, Filipovic MR, Alvarez B (2015). Reaction of hydrogen sulfide with disulfide and sulfenic acid to form the strongly nucleophilic persulfide. J. Biol. Chem..

[48] Yan W, Stone E, Zhang YJ (2017). Structural snapshots of an engineered cystathionine-γ-lyase reveal the critical role of electrostatic interactions in the active site. Biochemistry.

[49] Hopwood EM, Ahmed D, Aitken SM (2014). A role for glutamate-333 of *Saccharomyces cerevisiae* cystathionine γ-lyase as a determinant of specificity. Biochim. Biophys. Acta.

[50] Otwinowski Z, Minor W (1997). Processing of X-ray diffraction data collected in oscillation mode. Methods Enzymol..

[51] programs for protein crystallography (1994). Collaborative Computational Project Number 4. The CCP4 suite. Acta Crystallogr. Sect. D.

[52] Adams PD, Afonine PV, Bunkóczi G, Chen VB, Davis IW, Echols N, Headd JJ, Hung LW, Kapral GJ, Grosse-Kunstleve RW, McCoy AJ, Moriarty NW, Oeffner R, Read RJ, Richardson DC, Richardson JS, Terwilliger TC, Zwart PH (2010). PHENIX: a comprehensive Python-based system for macromolecular structure solution. Acta Crystallogr. D Biol. Crystallogr..

[53] Brünger AT (1992). The free *R* value: a novel statistical quantity for assessing the accuracy of crystal structures. Nature.

[54] Emsley P, Cowtan K (2004). Coot: model-building tools for molecular graphics. Acta Crystallogr. D Biol. Crystallogr..

[55] DeLano WL (2002). The PyMOL User’s Manual.

