# Supplementary material for: Catalytic specificity of the Lactobacillus plantarum cystathionine γ-lyase presumed by the crystallographic analysis
Source: Sci Rep. 2020 Sep 10;10:14886. doi: 10.1038/s41598-020-71756-7 (PMC7483736; doi:10.1038/s41598-020-71756-7)
Supplement: Supplementary file 1 — Supplementary Information. [file 41598_2020_71756_MOESM1_ESM.docx]

**Catalytic specificity of the *Lactobacillus plantarum* cystathionine γ-lyase presumed by the crystallographic analysis**

**Yasuyuki Matoba, Masafumi Noda, Tomoki Yoshida, Kosuke Oda, Yuka Ezumi, Chiaki Yasutake, Hisae Izuhara-Kihara, Narandarai Danshiitsoodol, Takanori Kumagai & Masanori Sugiyama**

**Author information**

**Affiliations**

Faculty of Pharmacy, Yasuda Women's University, Yasuhigashi 6-13-1, Asaminami-ku, Hiroshima 731-0153, Japan

Yasuyuki Matoba & Yuka Ezumi

Graduate School of Biomedical and Health Sciences, Hiroshima University, Kasumi 1-2-3, Minami-ku, Hiroshima 734-8551, Japan

Masafumi Noda, Tomoki Yoshida, Kosuke Oda, Hisae Izuhara-Kihara, Narandarai Danshiitsoodol, Takanori Kumagai & Masanori Sugiyama

Faculty of Pharmaceutical Sciences, Hiroshima University, Kasumi 1-2-3, Minami-ku, Hiroshima 734-8551, Japan

Chiaki Yasutake

**Supplementary Results**

**The catalytic activity of the *L. plantarum* CBS to generate L-cysteine perdulfide**

We have previously demonstrated that the *L. plantarum* CBS generates H_2_S in accordance with the condensation of two L-cysteine molecules (**Reaction 2** in **Supplementary Fig. S1**) or in accordance with the synthesis of cystathionine from L-cysteine and L-homocysteine (**Reaction 3** in **Supplementary Fig. S1**)^1^. In the present study, by using the cyanolysis method^2^, the catalytic activity of the *L. plantarum* CBS to generate L-cysteine persulfide from L-cystine was investigated in the presence or absence of L-homocysteine (**Supplementary Table S1**), demonstrating that the *L. plantarum* CBS generates L-cysteine persulfide in the presence of L-homocysteine more efficiently than in the absence of L-homocysteine. However, based on the *k*_cat_/*K*_m_ values, the catalytic efficiency of CBS measured in the presence of L-homocysteine was lower than that of CGL measured without L-homocysteine (**Table 1**).

**Supplementary Materials and Methods**

**Preparation of the *L. plantarum* CBS**

Expression and purification of the *L. plantarum* CBS were done according to the previous method^1^.

**Kinetic analysis**

The *L. plantarum* CBS may catalyze the generation of L-cysteine persulfide from L-cystine, because the concentration of the sulfane sulfur atom, which is included in L-cysteine persulfide but not in L-cystine, was increased in the reaction mixture. The main by-products are likely to be pyruvate and cystathionine in the absence and the presence of L-homocysteine, respectively (**Reactions 6 and 4** in **Supplementary Fig. S1**, respectively). The concentration of the sulfane sulfur in the reaction mixture was directly determined by the cyanolysis method^2^ to measure the enzymatic activity of the *L. plantarum* CBS to generate L-cysteine persulfide. The reaction mixture (1 mL), which consists of 100 mM HEPES-KOH (pH 7.5), 1.0–8.0 mM L-cystine, 0–10 mM L-homocysteine, and 25 μg mL^-1^ CBS, was incubated at 37°C for 20 min. The mixture without the CBS enzyme was used as a control. After the reaction was stopped by the addition of 40 μL each of 2 M ammonium hydroxide and 1.25 M potassium cyanide to the mixture, the incubation was continued at room temperature for 30 min. Then, 20 μL of 38% (v/v) formaldehyde and 200 μL of Goldstein’s reagent were added to the mixture. After centrifugation of the reaction mixture, the absorbance at 460 nm of the resulting supernatant was measured. A calibration curve was prepared using sodium thiocyanate at the known concentrations (0–0.15 mM).

In general, the CBS catalyzes a two-substrate reaction with a ping-pong mechanism^3,4^. In the case of the generation of L-cysteine persulfide in the absence of L-homocysteine, the initial rates of reaction at different substrate concentrations were expressed by single-substrate Michaelis–Menten equation (**Eq. 1**). In the case of the generation of L-cysteine persulfide in the presence of L-homocysteine, the initial reaction rates at different substrate concentrations were expressed by **Supplementary Equation S1**.

$v=\frac{k_{\mathrm{cat}}\cdot E_{t}\cdot S_{A}\cdot S_{B}}{K_{m}^{B}\cdot S_{A}+K_{m}^{A}\cdot S_{B}+S_{A}\cdot S_{B}}$ (**Supplementary Eq. S1**)

In **Supplementary Eq. S1**, S_A_ and S_B_ are the concentrations of the first substrate (L-cystine) and the second substrate (L-homocysteine), respectively. The kinetic parameters were evaluated by the non-linear least square method.

**Supplementary References**

1. Matoba, Y., Yoshida, T., Izuhara-Kihara, H., Noda, M. & Sugiyama, M. Crystallographic and mutational analyses of cystathionine β-synthase in the H_2_S-synthetic gene cluster in *Lactobacillus plantarum*. *Protein Sci.* **26**, 763–783 (2017).

2. Wood, J. L. Sulfane sulfur. *Methods Enzymol.* **143**, 25–29 (1987).

3. Miles, E. W. & Kraus, J. P. Cystathionine β-synthase: structure, function, regulation, and location of homocystinuria-causing mutations*. J. Biol. Chem.* **279**, 29871–29874 (2004).

4. Aitken, S. M. & Kirsch, J. F. The enzymology of cystathionine biosynthesis: strategies for the control of substrate and reaction specificity. *Arch. Biochem. Biophys.* **433**, 166–175 (2005).

**Supplementary Table S1. Kinetic parameters the of the *L. plantarum* CBS to generate L-cysteine persulfide**

| Parameters |  |
| --- | --- |
| **From L-cystine** | |
| *k*_cat_ (s^-1^) | 0.070 ± 0.002 |
| *K*_m_ for L-cystine (mM) | 3.2 ± 0.2 |
| *k*_cat_*/K*_m_ for L-cystine (mM^-1^ s^-1^) | 0.022 ± 0.002 |
| **From L-cystine and L-homocysteine** | |
| *k*_cat_ (s^-1^) | 0.60 ± 0.12 |
| *K*_m_ for L-cystine (mM) | 12 ± 3 |
| *K*_m_ for L-homocysteine (mM) | 1.2 ± 0.3 |
| *k*_cat_*/K*_m_ for L-cystine (mM^-1^ s^-1^) | 0.050 ± 0.017 |
| *k*_cat_*/K*_m_ for L-homocysteine (mM^-1^ s^-1^) | 0.51 ± 0.17 |

**

Supplementary Figure S1**. Possible alternative reactivities of CBS and CGL enzymes from *L. plantarum*. In addition to the L-OAS-dependent CBS reaction, *L. plantarum* CBS catalyzes the OASS reaction (**Reaction 1**), the condensation of two L-cysteine molecules coupled with the generations of H_2_S and lanthionine (**Reaction 2**), L-cysteine-dependent CBS reaction coupled with the generation of H_2_S (**Reaction 3**), L-cystine-dependent CBS reaction coupled with the generation of L-cysteine persulfide (**Reaction 4**), and L-cystine β-lyase reaction coupled with the generation of L-cysteine persulfide (**Reaction 6**). Differently from the other CBS enzymes, *L. plantarum* CBS cannot catalyzes L-cysteine β-lyase reaction coupled with the generation of H_2_S (**Reaction 5**)^1^. In addition to the CGL reaction, *L. plantarum* CGL catalyzes the L-cysteine β-lyase reaction (**Reaction 5**), L-cystine β-lyase reaction coupled with the generation of L-cysteine persulfide (**Reaction 6**), CBL reaction (**Reaction 7**), and L-homocysteine γ-lyase reaction coupled with the generation of H_2_S (**Reaction 8**). The figure was drawn by ChemDraw (CambridgeSoft).

**

 Supplementary Figure S2**. Ideal conformations of the intermediates (**IIIa**, **IVa**, and **Va**) formed during the γ-lyase reaction. In the upper representations looking along the bond between Cα and Cβ atoms, the latter atom overlaps the former one. Similarly, in the lower representations looking along the bond between Cβ and Cγ atoms, the latter atom overlaps the former one. A hydrogen atom attached to the Cα atom (shaded by pale gray) is moved to the lysine residue, whereas a hydrogen atom attached to the Cβ atom (shaded by dark gray) is moved to the C4’ atom in PLP. The figure was drawn by ChemDraw.

**

**

**Supplementary Figure S3**. Ideal conformations of the intermediates (**IIIb** and **IVb**) formed during the β-lyase reaction. In the representations looking along the bond between Cα and Cβ atoms, the latter atom overlaps the former one. A hydrogen atom attached to the Cα atom (shaded by pale gray) is moved to the lysine residue. The figure was drawn by ChemDraw.

**

Supplementary Figure S4.** Proposed mechanism underlying proton transfer from the lysine residue (K194^A^) at the active site to the tyrosine residue (Y97^A^) after the formation of **VIIIa** intermediate in the γ-lyase reaction or after the formation of **Vb** intermediate in the β-lyase reaction. *K* and *Y* in the figure mean K194^A^ and Y97^A^, respectively. The figure was drawn by ChemDraw.

**

Supplementary Figure S5**. Reactions interrupted by the interaction with the E320^A^ residue. **(a)**, L-cysteine β-lyase reaction. **(b)**, L-homocysteine γ-lyase reaction. Both reactions seem to proceed, after the hydrogen-bonding interaction between the substrate and the Y97^A^ residue is formed. The figure was drawn by ChemDraw.
